# Supplementary material for: Pragmatic cluster randomised cohort cross-over trial to determine the effectiveness of bridging from emergency to regular contraception: the Bridge-It study protocol
Source: BMJ Open. 2019 Oct 30;9(10):e029978. doi: 10.1136/bmjopen-2019-029978 (PMC6830607; doi:10.1136/bmjopen-2019-029978)
Supplement: Author's manuscript [file bmjopen-2019-029978.draft_revisions.pdf]

BMJ Open

BMJ Open is committed to open peer review. As part of this commitment we make the peer review history of every article we publish publicly available.

When an article is published we post the peer reviewers' comments and the authors' responses online. We also post the versions of the paper that were used during peer review. These are the versions that the peer review comments apply to.

The versions of the paper that follow are the versions that were submitted during the peer review process. They are not the versions of record or the final published versions. They should not be cited or distributed as the published version of this manuscript.

BMJ Open is an open access journal and the full, final, typeset and author-corrected version of record of the manuscript is available on our site with no access controls, subscription charges or pay-per-view fees (http://bmjopen.bmj.com).

If you have any questions on BMJ Open's open peer review process please email info.bmjopen@bmj.com

BMJ Open

A cluster randomised controlled trial to determine the effectiveness of bridging from emergency to regular contraception: The Bridge–It study protocol

Journal:	*BMJ Open*
Manuscript ID	bmjopen-2019-029978
Article Type:	Protocol
Date Submitted by the Author:	16-Apr-2019
Complete List of Authors:	Cameron, Sharon; University of Edinburgh, Obstetrics and Gynaecology; NHS Lothian, Sexual and Reproductive Health<br>Baraitser, Paula; King's College London School of Medical Education<br>Glasier, Anna; University of Edinburgh<br>McDaid, Lisa; University of Glasgow, MRC/CSO Social and Public Health Sciences Unit<br>Norrie, John; Edinburgh Clinical Trials Unit, University of Edinburgh No. 9, Bioquarter<br>Radley, Andrew; NHS Tayside, Directorate of Public Health; University of Dundee Division of Cardiovascular and Diabetes Medicine, Liver Group<br>Stephenson, Judith; University College London, Instiute for Women's Health<br>Trussell, James; Princeton university, Office of Population Research<br>Battison, Claire; Edinburgh Clinical Trials Unit, University of Edinburgh No. 9, Bioquarter<br>Cameron, Sarah; University of Aberdeen, Health Services Research Unit<br>Cowle, Kathleen; Boots UK Ltd<br>Forrest, Mark; University of Aberdeen, Health Services Research Unit<br>Gilson, Richard; University College London, Institute for Global Health<br>Goulao, Beatriz; University of Aberdeen, Health Services Research Unit<br>Johnstone, Anne; University of Edinburgh, Obstetrics and Gynaecology<br>McDonald, Alison; University of Aberdeen, Health Services Research Unit<br>Morelli, Alessandra; King's College London School of Medical Education<br>Patterson, Susan; University of Glasgow, MRC/CSO Social and Public Health Sciences Unit<br>Sally, Deirdre; University College London, Instiute for Women's Health<br>Stewart, Nicola; University College London, Instiute for Women's Health
Keywords:	contraception, emergency contraception, pharmacy, unintended pregnancy, levonorgestrel, progestogen only pill

A cluster randomised controlled trial to determine the effectiveness of bridging from emergency to regular contraception: The Bridge–It study protocol

Bridge-It Study Group*

***Sharon Cameron[1], Paula Baraitser [2], Anna Glasier [1], Lisa McDaid [3], John Norrie [4], Andrew Radley [5], Judith Stephenson [6], James Trussell [7†],Claire Battison [4], Sarah Cameron [8] , Kathleen Cowle [9], Mark Forrest [8], Richard Gilson [10], Beatriz Goulao [8], Anne Johnstone [1], Alison McDonald [8], Alessandra Morelli [11], Susan Patterson [3], Deirdre Sally [6], Nicola Stewart [6]**

[1]Corresponding author: Professor Sharon Cameron, Consultant Gynaecologist, Address: 2a Chalmers Street, Edinburgh, EH3 9ESTel: 00 44 131 536 2091 Email: sharon.cameron@ed.ac.uk

Word Count: 3767

Funder: HTA/NIHR
Funding Reference Number: HTA Project 15/113/01

In addition, the following disclaimer should be included in this paper:

The views expressed are those of the author(s) and not necessarily those of the NIHR, the NHS or the Department of Health

For peer review only - http://bmjopen.bmj.com/site/about/guidelines.xhtml

ABSTRACT:

Introduction: Oral emergency contraception (EC) can prevent unintended pregnancy but it is important to start a regular method of contraception. Women in the UK usually access EC from a pharmacy but then need a subsequent appointment with a GP or a sexual and reproductive health (SRH) service to access regular contraception. Unintended pregnancies can occur during this time.

Methods and analysis: Bridge-It is a pragmatic cluster randomised cohort crossover trial designed to determine whether pharmacist provision of a bridging supply of a progestogen only pill (POP) plus rapid access to a local SRH clinic, results in increased uptake of effective contraception and prevents more unintended pregnancies than provision of EC alone. Bridge-It involves 31 pharmacies in three UK regions (London, Lothian and Tayside) aiming to recruit 626 to 737 women. Pharmacies will give EC (levonorgestrel) according to normal practice and recruit women to both intervention and the control phases of the study. In the intervention phase, pharmacists will provide the POP (desogestrel) and offer rapid access to a SRH clinic. In the control phase, pharmacists will advise women to attend a contraceptive provider for contraception (standard care).

Women will be asked four months later about contraceptive use. Data linkage to abortion registries will provide abortion rates over 12 months. The sample size is calculated on the primary outcome of effective contraception use at four months (yes/no) with 90% power and a 5% level of significance. Abortion rates will be an exploratory secondary analysis. Process evaluation includes interviews with pharmacists, SRH clinicians and women. Cost-effectiveness analysis will use a healthcare system perspective and be expressed as incremental cost-effectiveness ratio.

Ethics and dissemination: Ethical approval was received from South East Scotland REC June 2017. Results will be published in peer-reviewed journals and conference presentations.

Trial registration number ISRCTN 70616901

Strengths and limitations:

- Innovative and efficient cluster cohort crossover design
- Examines impact of the intervention on uptake of effective contraception at four months
- Examines the important outcome of abortion rates over 1 year as an exploratory secondary analysis.
- Applicable only to women receiving levonogrestrel EC followed by a desogestrel POP
- Not applicable to use of ulipristal acetate for EC, since hormonal methods of contraception such as the desogestrel POP may interact with efficacy of ulipristal acetate, if started within five days

For peer review only - http://bmjopen.bmj.com/site/about/guidelines.xhtml

INTRODUCTION

Unintended pregnancy is a major public health problem. The UK has among the highest abortion rates in Europe [1]. In 2017 almost 200,000 pregnancies ended in induced abortion [2, 3]. Unintended pregnancy also ends in childbirth; around 10% of UK births are unintended and 25% mistimed [4]. Unintended pregnancy is costly to the NHS (estimated to cost over £1 billion annually) [5] and can be distressing for women. Unintended pregnancies are more common in young women from deprived backgrounds, contributing to widening health inequalities for both mother and baby, and their families [2,3]. Unintended childbirth can have both socioeconomic consequences for women and their families and mental health consequences [6].

Oral emergency contraception (EC) prevents pregnancy in individual women following unprotected sex or contraceptive accidents. EC is only effective if taken before ovulation as it works by inhibiting or delaying ovulation [7]. Since EC became available from pharmacies in the UK without the need for a prescription, there has been a change in the pattern of access such that women who seek EC now choose to obtain this from a pharmacy rather than a contraceptive provider such as a GP or sexual and reproductive health (SRH) service [8]. Although trials have shown that this facilitates access to EC and increases use, they have failed to show that this reduces unintended pregnancy rates within the population [9].

There are two types of EC: the most widely used EC contains the progestogen levonorgestrel and should be taken within 72 hours of sex; the other EC contains the progesterone receptor modulator ulipristal acetate and should be taken within 120 hours of sex [10]. Neither formulation of EC prevents conceptions from subsequent acts of sex and the risk of pregnancy is increased up to threefold among women who have further unprotected sex in the same menstrual cycle after using EC than those who do not [10]. An effective method of contraception should therefore be started as soon as possible [10, 11]. However the only contraceptives that can be obtained from any pharmacy without a prescription are condoms, which have high failure rates [12]. This means that women usually need to make an appointment with a contraceptive provider (GP or SRH) and may experience delays in accessing regular contraception or lose the motivation to access a regular method altogether, which in turn may result in unintended pregnancies. In addition, in one UK study fewer than half of pharmacists gave advice about ongoing contraception after EC [13]. It is possible that if pharmacists could supply a temporary (bridging) method of contraception to women along with EC, this would bridge the gap until women could get an appointment with a contraceptive provider for contraceptive advice and supplies. The progestogen only pill (POP) is an effective method of contraception with few contraindications [14] making it safer than the combined oral contraceptive pill for pharmacy provision. However, studies have shown that starting hormonal contraception containing a progestogen within five days of ulipristal acetate may reduce the efficacy of EC and so only EC containing levonorgestrel is suitable for use in conjunction with a bridging method of hormonal contraception in this way [15, 16].

For peer review only - http://bmjopen.bmj.com/site/about/guidelines.xhtml

Pilot

In a pilot study in Edinburgh of 168 women presenting for EC [17] 11 pharmacies were randomised to one of three groups to provide EC (levonorgestrel) and either (i) standard advice on where to obtain ongoing contraception or (ii) one month of a progestogen only pill (POP) or (iii) the offer of rapid access to a local SRH service. Participants were contacted by telephone six to eight weeks later to determine their current contraceptive use. Compared to standard care, the proportion of women using effective contraception was significantly greater in both the POP (56% vs. 16% p=0.001) and the rapid access groups (52% vs. 16% p=0.027). This suggests that a supply of one month of POP after EC or rapid access from a pharmacy to a SRH service might increase short-term uptake of effective contraception following EC. We now propose a large randomised trial to determine whether a pharmacy-based intervention designed to facilitate the uptake of effective contraception after EC increases use of effective contraceptive methods including the most effective long acting reversible contraceptive methods (LARC) such as the contraceptive implant and intrauterine contraception[12] at four months when compared with standard care. We will examine contraceptive uptake at four months as most POP preparations are packaged as a three month supply and so by four months the pharmacy provided supply will have ended.

Aim

The aim is to develop a simple and affordable intervention which facilitates the uptake of effective ongoing contraception among women obtaining EC from pharmacies thereby reducing unintended pregnancy. The primary objective is to determine whether offering women attending a pharmacy for EC, a three month bridging supply of POP plus the offer of rapid access to a local SRH service results in increased uptake of effective contraception. This combined intervention (POP plus rapid access) offers both temporary contraception and facilitates access to a specialist contraceptive service where all methods of contraception including the most effective LARC methods can be provided. If this intervention leads to increased uptake of effective contraception including LARC methods compared to standard care alone then we might expect that this would translate into fewer unintended pregnancies for women.

METHODS AND ANALYSIS

Study design and setting

A pragmatic cluster randomised cohort crossover clinical and cost effectiveness trial including process, outcome and economic evaluation involving 31 pharmacies in three UK regions (15 in London (South and Central), 12 in Lothian (Edinburgh and region) and four in Tayside (Dundee and region).

Patient and Public Involvement

The members of the Patient and Public Involvement (PPI) Group at the SRH service in Edinburgh contributed to the design of this study. The study protocol and documentation were reviewed and approved by the chair and members of the PPI group. The plain English summary was edited by a PPI member and improved as a result. There are three PPI members that participate and contribute to the Bridge-It

For peer review only - http://bmjopen.bmj.com/site/about/guidelines.xhtml

Trial steering Committee meeting (TSC) that provides oversight of the study. PPI group will be involved in the dissemination of the study results.

Intervention

The planned intervention is a composite intervention. Each woman in the intervention phase will receive three months of POP (in a single package covering 3 months) and an invitation to attend a local participating SRH service to discuss and obtain effective contraception (including LARC methods). Three packets of POP, (75 mcg desogestrel; UK) containing 28 tablets each will be provided at no cost to women as a bridging method of contraception, providing them with three months of contraception during which time they can get an appointment with a contraceptive provider to obtain their preferred method of contraception. Locally approved Patient Group Directions (i.e. strict criteria to permit provision of specified medicines by non-prescribers) will permit participating pharmacists to dispense the POP to women recruited to the study. Pre-study training will be undertaken with participating pharmacists including identifying medical contraindications to POP, potential drug interactions medications and 'missed pill' guidance for POP. Pharmacists will advise women to start the POP the day following intake of EC [10].

Pharmacists will encourage women to attend the participating local SRH service to obtain the contraceptive method of their choice. Participants (intervention phase) will be given a study card to alert staff at SRH services that they are in the Bridge-It study and should be seen as a drop in for contraception that same day. This card will also provide written information about the location and opening hours of the local participating SRH service. These SRH services are within a 5-mile radius of the participating pharmacies and provide all methods of contraception at no cost as is the norm in the NHS.

Standard Care

A mystery shopper exercise [13] will be undertaken in all 31 participating pharmacies to characterise 'standard care' (usually verbal advice to visit a clinic for contraception, with/without written information) in the control phase. The mystery shopper visits will be conducted when the pharmacy is not recruiting and just before the control phase starts. The mystery shoppers and the scenario used will be chosen by the Patient Public Involvement group. A simple scenario relating to request for EC will be used. Immediately after leaving the pharmacy the mystery shopper will complete a standard data collection proforma, recording any information given by the pharmacist about use of contraception after taking EC, including provision of written information.

Participants

We will recruit a total of 626 to 737 women presenting for EC. The final number will be determined based on the observed ratio of the between-period within-cluster variability (BPC) and the within-period within-cluster (WPC) variability – with the larger sample size (near the 737 upper limit) required for values of BPC close to zero, and the smaller sample size (near the 626 lower limits) required for values of BPC close to WPC (18). Each pharmacy will be expected to recruit an average of 12-13 women to the intervention arm and 12-13 women to standard care. This allows for a 25% loss to follow up at four months (missing data on primary outcome).

For peer review only - http://bmjopen.bmj.com/site/about/guidelines.xhtml

Randomisation

Each pharmacy will be randomised to either the intervention phase for approximately 20 weeks followed by standard care phase for 20 weeks or vice-versa with a wash-out period of two weeks between the two phases. The order in which pharmacy allocation to each arm is undertaken (intervention or control first) will be randomised (Figure 1).

This is a cluster cohort crossover design so it is the pharmacy that is the unit of randomisation and the 'crossover' means that we are just randomising the order that each pharmacy gives the intervention in. The ‘cohort’ label means that we expect different women to be recruited within each site in the two periods (intervention and control phases).

Recruitment

The pharmacist will assess medical eligibility of women presenting for EC for the study, provide EC according to normal practice and invite eligible women to participate. The EC used in this study is levonorgestrel and will be given in the clinically indicated dose for the woman’s weight (1.5 mg or 3 mg levonorgestrel) [10].

Inclusion and exclusion criteria are shown in Table 1. Women who give written consent will be recruited in the study. We recognise the importance of participant retention and will offer a voucher of £10 at recruitment [19].

Outcomes

A full list of study outcome measures is included in Table 2. Outcomes at four months will primarily be collected via telephone interviews or via web based questionnaires. However, participants will also have the option to provide the same information by a postal questionnaire. The primary outcome is use of effective contraception at four months (intervention vs standard care).

Secondary outcomes are proportion of participants having an abortion within 12 months of EC use using record linkage from participants to national registries and cost effectiveness.

Process evaluation measures

A process evaluation will be conducted as part of the study to assess potential issues concerning intervention implementation, the causal mechanisms of impact, and the contextual factors that could affect these. The process evaluation will comprise of quantitative and qualitative data measures, as detailed in Table 3.

Data Collection

Quantitative data

Participant flow: Participant flow through the study will be assessed and reported following the CONSORT flow chart.

Baseline

Participant demographics and reproductive history is collected at recruitment by a self-administered paper questionnaire given to them by the pharmacist.

For peer review only - http://bmjopen.bmj.com/site/about/guidelines.xhtml

For peer review only

Demographic data will also be reported for the process evaluation, protocol adherence checklists and for recruitment screening forms (see Table 3).

Contraceptive use at 4 months
This will be based upon self-reported data from women at a telephone follow up interview with a research nurse at four months after obtaining EC. If participants prefer, the questions can be self-completed by a web based questionnaire or paper questionnaire sent by post. Women will be asked what method of contraception they are using (if any), if they attended a GP or SRH service for this, if they used the POP (intervention phase only) and their pregnancy status. If pregnancy has occurred since EC then the validated London Measure of Unintended Pregnancy tool will be administered to measure intended-ness of the index pregnancy [20] (Table 2).

Abortion rates at 12 months
Information Services Division (ISD Scotland) and Department of Health (DOH England) will provide the number of abortions occurring during the 12 month follow up period in each arm by conducting linkage of the identifiers (collected at baseline) from study participants.

Validation
We will check with data from local SRH services to determine the numbers of participants from intervention and control phases who attend the local participating SRH service, and which method of contraception they received.

Qualitative data
Semi-structured, qualitative telephone interviews of a purposive sample of up to 60 women who received the intervention (approximately 22 in London, 30 in Edinburgh and 8 in Dundee) will be undertaken. Participants who consent to these telephone interviews will be contacted by the Process Evaluation Research Assistant. Interviews will explore experience of intervention acceptability in more depth and assess experiences of bridging from EC to regular contraception, and reasons for doing so or not (Table 3). Interviews will be conducted soon after the four month follow up.

Interviews with 27 pharmacists and 12 SRH service providers will explore their perceptions of barriers and facilitators to implementation and more broadly, their views on the intervention, the trial, and the target population. Interviews will be conducted by the process evaluation research assistant soon after the intervention phase has completed.

For the process evaluation, data collection also includes: review of training and materials; observation of training; mapping of local contraceptive services within 10 miles of study sites and monitoring of contemporaneous events, such as relevant high coverage media stories using Google Alerts (Table 3).

Sample size calculation
The study is a cluster randomised cohort crossover trial. Ideally the control and intervention phases should be of roughly equal duration and size, and the sample size is calculated assuming an equal cluster size in both control and intervention

For peer review only - http://bmjopen.bmj.com/site/about/guidelines.xhtml

periods, and equal across sites (the expected site/period average). In practice, there is variability in EC demand across sites and over time: for example, demand is affected by peak holiday periods (recruitment decreases) and student term times (recruitment increases); and the ability of a pharmacy to translate that variable demand into study recruits depends on many factors, including changes to circumstances at individual pharmacies or loss of / change of pharmacists at multi-pharmacist stores.

Informed by our pilot study, we have assumed that effective contraception use in the control would be 30% and we were likely to achieve a 50% relative improvement to 45%. This means the sample size is in the range 626 to 737, assuming 25% of women do not provide four-month contraceptive use data, and an average cluster size of 12-13 in each period, and around 25 pharmacies taking part, with 90% power and a 5% level of significance. The uncertainty in the required sample size rests on the assumed between-period within-cluster correlation (BPC) and its relationship to the other component of variability, the within-period within-cluster correlation (WPC)[18]. The WPC is the usual correlation (known as the Intra Class Correlation–ICC in a standard parallel groups non-crossover cluster setting) of two individuals' outcomes within a cluster (in the same period). The BPC on the other hand is the correlation between two individuals' outcomes in the same cluster between the two periods. If the BPC is zero, there is no advantage in a crossover design over the standard parallel groups cluster design; if the BPC equals the WPC then the crossover is as efficient as an individually randomised design. We will finalise the sample size depending on the observed ratio of the BPC to WPC, and the observed rate of attrition, but still assuming the same control rate and treatment effect, once we have 4 month data on at least 500 participants.

Quantitative Analyses

There will be a single analysis at study end (there is no opportunity for any interim analyses given the crossover design) although an independent Data Monitoring Committee will monitor study progress and any safety issues. This will follow the intention to treat principle and will use a hierarchical model appropriate for the specific outcome. For the primary outcome this will be a mixed effects logistic regression, using the hierarchical model approach as recommended by Turner for a cluster crossover design [21]. We will pre-specify any individual level (or cluster level) covariates that we intend to adjust for, and the comprehensive Statistical Analysis Plan will specify the sensitivity type analyses that will explore how robust the findings are to any missing data at the cluster level (probably unlikely) and the individual level (expected to be substantial for the patient reported outcomes at four months). As well as the usual assumption of missing at random, we will try to explore possible mechanisms for non-ignorable (informative missingness) at the individual level which may well be operating in this context. Subgroup analyses (appropriately analysed by testing treatment by subgroup interaction) will explore the possible effect modification by LARC (most effective contraceptive methods) vs non-LARC.

Qualitative and mixed-methods process evaluation analyses

All process data will be analysed independently of the outcome data and, importantly, documented before the outcomes are known. Qualitative analysis of in -depth interviews will be recorded and transcribed verbatim. Transcription and

For peer review only - http://bmjopen.bmj.com/site/about/guidelines.xhtml

analysis (proceeding case by case) will start with the first interview and be ongoing during the course of data collection, allowing for emergent themes to be identified and explored in future interviews. The transcripts will be read repeatedly and coded for analysis. Data management will be assisted by the software, QSR NVivo 10. Analysis will be undertaken using 'Framework Analysis' a method of proven validity and reliability where data are coded, indexed and charted systematically, then organised using a matrix or framework [22]. Constant comparison will be carried out to ensure that the analysis represents all perspectives and negative ('deviant') cases.

The multi-source process evaluation will be synthesised to address the three key process evaluation questions: i) what was delivered, ii) how it was delivered, and iii) what role context may have had in shaping the delivery/outcomes

Economic evaluation: An economic evaluation will be undertaken comparing the intervention and control arms in a cost effectiveness analysis. A trial-based analysis will be followed by the construction of a decision model to extrapolate future costs and benefits beyond the completion of the trial. The overall perspective used will be that of the health system. Costs will include the pharmacist training to provide POP, direct and indirect costs of health service use, and the provision and dispensing of POP. We will compare the costs to the NHS in the intervention and control arms. To account for differences in the numbers of women in the two arms, we will compare the cost per woman in each arm. In the control arm, the costs are (i) cost of EC, (ii) cost of pharmacist provision of EC, (iii) cost of abortions. In the intervention arm, the costs are in addition to these (iv) the cost of the POP, (v) cost of pharmacist training to provide POP and (vi) cost of pharmacist provision of POP. The costs (i) and (ii) are the same in both groups and so the extra cost of the intervention will be the sum of (iv), (v) and (vi). The cost per women who has an abortion is the same in both groups except that we hypothesise that the abortion rate will be lower in the intervention group. We can then state the outcome as conventional incremental cost effectiveness ratio i.e. for every £100 spent on the intervention results in x fewer abortions for a savings of £Y. If Y is greater than 100 then the intervention is cost effective. We will examine the sensitivity of the outcomes to variations in the costs of iv, v, and vi.

Data Management and Clinical Trial Unit support

Data will be collected on a paper case report form and will be entered directly into the trial database. Data will be entered into a trial database by pharmacists, research nurses or staff at the trial co-ordinating centre. The data management and statistical support for the study is provided by the UKCRC registered Clinical Trial Unit (CTU) the Centre for Healthcare Randomised Trials (CHaRT) at the University of Aberdeen; while the trial management is provided by another UKCRC registered CTU, the Edinburgh Clinical Trials Unit (ECTU) at Edinburgh University.

Ethics and dissemination

The Bridge-It trial involves procedures and medications which are well established in current NHS clinical practice and use. Adverse events may occur during or after the use of EC or POP and are well documented in the POP patient information leaflet.

For peer review only - http://bmjopen.bmj.com/site/about/guidelines.xhtml

Serious adverse events will be recorded at the four month follow up interview. The study will be conducted in accordance with the principles of Good Clinical Practice (GCP).

A favorable ethical opinion has been obtained from the South East Scotland REC in June 2017. Approvals have been obtained from NHS Research Scotland (NRS) and Health Research Authority (HRA) England prior to commencement of the study. Annual progress reports and a final report at the conclusion of the trial will be submitted to REC within the timelines defined in the regulations.

The Bridge-It study website will include trial materials, trial progress, and summaries of key findings. In addition, public engagement and dissemination will also be undertaken via our Patient Public Involvement group.

The results of the study will be published in the academic journals and all participants will be offered a lay summary of the main findings of the study. The findings will also be presented at national and international conferences and disseminated via social media.

Trial Status

As at 7th Feb 2019 the study had recruited 503 participants across 29 sites. Recruitment to the first period completed on 13th Jan 2019, with 391 participants recruited at 29 sites. Recruitment is scheduled to be completed by Jun 2019, with analyses of the 4-month primary outcome expected to be available by Oct 2019.

DISCUSSION

Unintended pregnancy remains a major public health problem. The proposed intervention in the Bridge-It study provides both temporary contraception (the POP) and facilitates access to effective contraception at a local SRH clinic. With the cluster crossover design, each cluster will act as its own control and fewer pharmacies are required than with a parallel cluster design.

If our proposed intervention works, then this could prevent more unintended pregnancies for more women. If the intervention is cost effective then it could have cost savings for the NHS.

For peer review only - http://bmjopen.bmj.com/site/about/guidelines.xhtml

REFERENCES.

1. Sedgh G, Singh S, Shah IH et al. Induced abortion: incidence and trends worldwide from 1995 to 2008. *Lancet*. 2012Feb 18;379(9816):625-32.

2.Department of Health. Abortion Statistics, England and Wales: 2017. https://www.gov.uk/government/publications/report-on-abortion-statistics-in-england-and-wales-for-2014 (access date 11 Jan 2019)

3. Information Services Division. Termination of Pregnancy Statistics 2017 http://www.isdscotland.org/Health-Topics/Sexual-Health/Abortions/ (access date 11 Jan 2019)

4. Wellings K, Jones KG, Mercer CH et al. The prevalence of unplanned pregnancy and associated factors in Britain: findings from the third National Survey of Sexual Attitudes and Lifestyles (Natsal-3). *Lancet* 2013;382:1807-16

5. Thomas C, Cameron ST. Can we reduce costs and prevent more unintended pregnancies? A cost of illness study illustrating the cost-saving potential of a more effective method of EHC. *BMJ open* 2013;3:e003815.doi:10.1136

6. SmithBattle L, Leonard V. Inequities compounded: explaining variations in the transition to adulthood for teen mothers' offspring. *J Fam Nurs*. 2012;18(3):409-31.

7. Cameron ST, Li H R**,** Gemzell –Danielsson K. Current controversies with Emergency Contraception: a review. *BJOG* 2017(13):1948-1956.

8. Marston C, Meltzer H, Majeed A. Impact on contraceptive practice of making emergency hormonal contraception available over the counter in Great Britain: repeated cross sectional surveys.*BMJ*. 2005Jul 30;331(7511):271.

9. Polis CB, Grimes DA, Schaffer K, et al . Advance provision of emergency contraception for pregnancy prevention. Cochrane Database of Systematic Reviews 2007,Issue2.Art. No: CD005497. DOI:0.1002/14651858.CD005497.pub2.

10. Faculty of Sexual and Reproductive Healthcare, UK. Emergency contraception. 2017. www.fsrh.org (access date 11 January 2019)

11. Cheng L, Che Y, Gülmezoglu AM. Interventions for emergency contraception. *Cochrane Database of Systematic Reviews* 2012;Issue 8. Art. No.: CD001324. DOI: 10.1002/14651858.CD001324.pub4.

12. Trussell J. Contraceptive efficacy. Global Library of Womens Medicinehttp://www.glowm.com/section_view/heading/Contraceptive%20Efficacy/item/374 (access date 29 Jan 2019)

For peer review only - http://bmjopen.bmj.com/site/about/guidelines.xhtml

13 .Glasier A, Manners RJ, Loudon JC, et al. Community pharmacists providing emergency contraception give little advice about future contraceptive use: a mystery shopper study. *Contraception* 2010;82: 538–42.

14. The Faculty of Sexual and Reproductive Healthcare (FSRH). UK medical Eligibility criteria for Contraceptive Use. http://www.fsrh.org/standards-and-guidance/uk-medical-eligibility-criteria-for-contraceptive-use/ (access date 11 Jan 2019)

15. Brache V, Cochon L, Duijkers IJ, et al. A prospective, randomized, pharmacodynamic study of quick-starting a desogestrel progestin-only pill following ulipristal acetate for emergency contraception. *Hum Reprod* 2015;30(12):2785-2793

16. Cameron ST, Berger C, Michie L,et al. The effects on ovarian activity of 'quickstarting' a combined oral contraceptive pill after ulipristal acetate : prospective, randomised, double-blind parallel-arm, placebo-controlled study. *Hum Reprod* 2015;30(7):1566-1572.

17. Michie L, Cameron ST, Glasier A et al .Pharmacy based interventions for initiating effective contraception following the use of emergency contraception: a pilot study. *Contraception* 2014 ;90, 447-53

18. Arnup SJ, McKenzie JE, Hemming K, et al. Understanding the cluster crossover design: a graphical illustration of the components of variation and a sample size tutorial. *Trials.* 2017;18; 381.

19. Brueton VC, Tierney J, Stenning S, et al. Strategies to improve retention in randomised trials. Cochrane Database Syst Rev. 2013 Dec 3; 12:MR000032. doi: 10.1002/14651858.MR000032.pub2. 21

20. Cameron ST ,Glasier A. Identifying women in need of further discussion about the decision to have an abortion and eventual outcome. *Contraception*. 2013**;**88(1): 128-32

21. Turner RM, Omar RZ, Thompson SG. Modelling multivariate outcomes in hierarchical data, with application to cluster randomised trials. *Biom J*. 2006 Jun;48(3):333-45.

22. Ritchie J, Spencer L. Qualitative research practice: a guide for social science students and researchers. Sage: London, 2003.

Authors' contributions:

STC, AG, JN, LMc D, AR, PB, JS, JT developed the original protocol. CB contributed to later stages of study design. STC wrote the draft of the protocol with significant input from all authors at all stages. All authors contributed, read and approved the final manuscript.

For peer review only - http://bmjopen.bmj.com/site/about/guidelines.xhtml

Author affiliations

1. Chalmers Centre, NHS Lothian, Edinburgh EH3 9ES and University of Edinburgh
2. King's College Hospital NHS Foundation Trust, London, SE5 9RS
3. MRC/CSO Social & Public Health Sciences Unit, University of Glasgow, Glasgow G2 3AX
4. Edinburgh Clinical Trial Unit, Usher Institute, University of Edinburgh, Edinburgh EH16 4UX
5. King's Cross Hospital, NHS Tayside, Dundee DD3 8EA
6. University College London, EGA Institute for Women's Health, London WC1E 6AU
7. †Deceased December 2018. Princeton University USA.
8. Health Service Research Unit, University of Aberdeen, Aberdeen, AB25 2ZD
9. Boots UK, North Hub Office, Falkirk FK1 1ES
10. University College London, Institute for Global Health, London WC1E 6JB
11. King's College London, SE5 9RJ

Acknowledgements

Thank you to the members of the Patient and Public Involvement Group at the SRH service in Edinburgh who contributed to the design of this study. Thanks to the women who have participated in this study and to the community pharmacists, research staff and health care professionals at the local SRH services who have assisted with the implementation of this study. Thanks to Laura Flett for trial management support.

The Bridge-It Study Steering Committee provides oversight for this trial on behalf of the sponsor (University of Edinburgh and NHS Lothian) and the funder. The members are: Professor Peter Brocklehurst (Chair), Birmingham Clinical Trials Unit; Dr Lucy Michie, Sandyford Glasgow; Professor Kaye Wellings, London School of Hygiene and Tropical Medicine; Joanna Loudon, PPI member, Edinburgh; Kirsten Stuart PPI member Edinburgh; Emily Whittaker PPI member Edinburgh.
The Data Monitoring Committee is an independent multidisciplinary group consisting of clinicians and statisticians. The members are: Professor Claire Anderson (Chair), University of Nottingham; Professor Elizabeth Allen, London School of Hygiene and Tropical Medicine; Professor Caroline Moreau, Johns Hopkins Bloomberg School of Public Health, USA.

The paper presents independent research funded by the National Institute for Health Research (NIHR). The views expressed are those of the authors and not necessarily those of the NHS, the NIHR or the Department of Health.

Funding statement: The Bridge-It study is funded by the National Institute for Health Research's Health Technology Assessment Programme. HTA Project:15/113/01

For peer review only - http://bmjopen.bmj.com/site/about/guidelines.xhtml

Competing interests statement.

AG is a member of HRA Pharma scientific advisory board.

PB is a Clinical Director of the not-for profit community interest company SH:24 that provides online sexual health services in partnership with the NHS.

AR Research grants, educational grants and consultancy with Gilead
Research grants from Roche and BMS
Educational grants from Abbvie

JN is Deputy Chair of the NIHR/HTA General Board Committee. NIHR/HTA funded this research.

For peer review only

For peer review only - http://bmjopen.bmj.com/site/about/guidelines.xhtml

Table 1. Inclusion and exclusion criteria

**Inclusion Criteria**<br>• Intake of EC (levonorgestrel)<br>• Capacity to give informed consent to participate in the trial which includes adherence to trial requirements<br>• Age 16 years or over<br>• Willing to give contact details and be contacted at four months by phone or text or e-mail or post<br>• Willing to give identifying data sufficient to allow data linkage with NHS registries
**Exclusion Criteria**<br>• Contraindications to the POP<br>• On medication that interacts adversely with POP<br>• Already using a hormonal method of contraception<br>• Require interpreting services<br>• If pharmacist has concerns about non-consensual sex

For peer review only

Table 2. Study outcomes

		Data Source
Main outcome	Use of effective contraceptive method (hormonal or intrauterine) in intervention vs control at 4 months	Self reported (telephone or self completed survey) at 4 months
Secondary Outcomes	Numbers undergoing an abortion within 12 months intervention vs control	National abortion registries
	Economic evaluation	Incremental cost effectiveness ratio for pregnancies prevented

For peer review only

For peer review only - http://bmjopen.bmj.com/site/about/guidelines.xhtml

Table 3: Process evaluation methods and data

**Theory**
Theory of change model
**Study Team**
Pharmacy recruitment forms (study team members involved in recruitment will routinely record decision making contributing to pharmacy selection, including: number of contacts made; responses from potential pharmacists; rationales for inclusion/exclusion; and reasons for refusal)
**Pharmacists**
Participant observation of training & review of training and intervention materials<br><br>Recruitment monitoring forms (n=100% of pharmacists) & protocol adherence checklists (n=100% of pharmacists)<br><br>Follow-up semi-structured telephone interviews with pharmacists (n=27; one with each pharmacy involved).
**SRH Providers**
Semi-structured telephone interviews with SRH providers (n=12; with Service Manager, mix staff at 2x services in London; 1x service in Edinburgh; 1x service in Dundee).
**Participants**
Telephone questionnaire administered by Research Nurse at 4 months post-intervention (n=100% participants)<br><br>Semi-structured telephone interviews at 4 months post-intervention (n=60; 22 in London, 30 in Edinburgh and 8 in Dundee)
**Context**
Audit of local contraceptive services within 10 miles of study sites in London, Edinburgh and Dundee<br><br>Monitoring of contemporaneous events, such as relevant high coverage media stories using Google Alerts

For peer review only - http://bmjopen.bmj.com/site/about/guidelines.xhtml

Figure 1: Bridge-It study flowchart

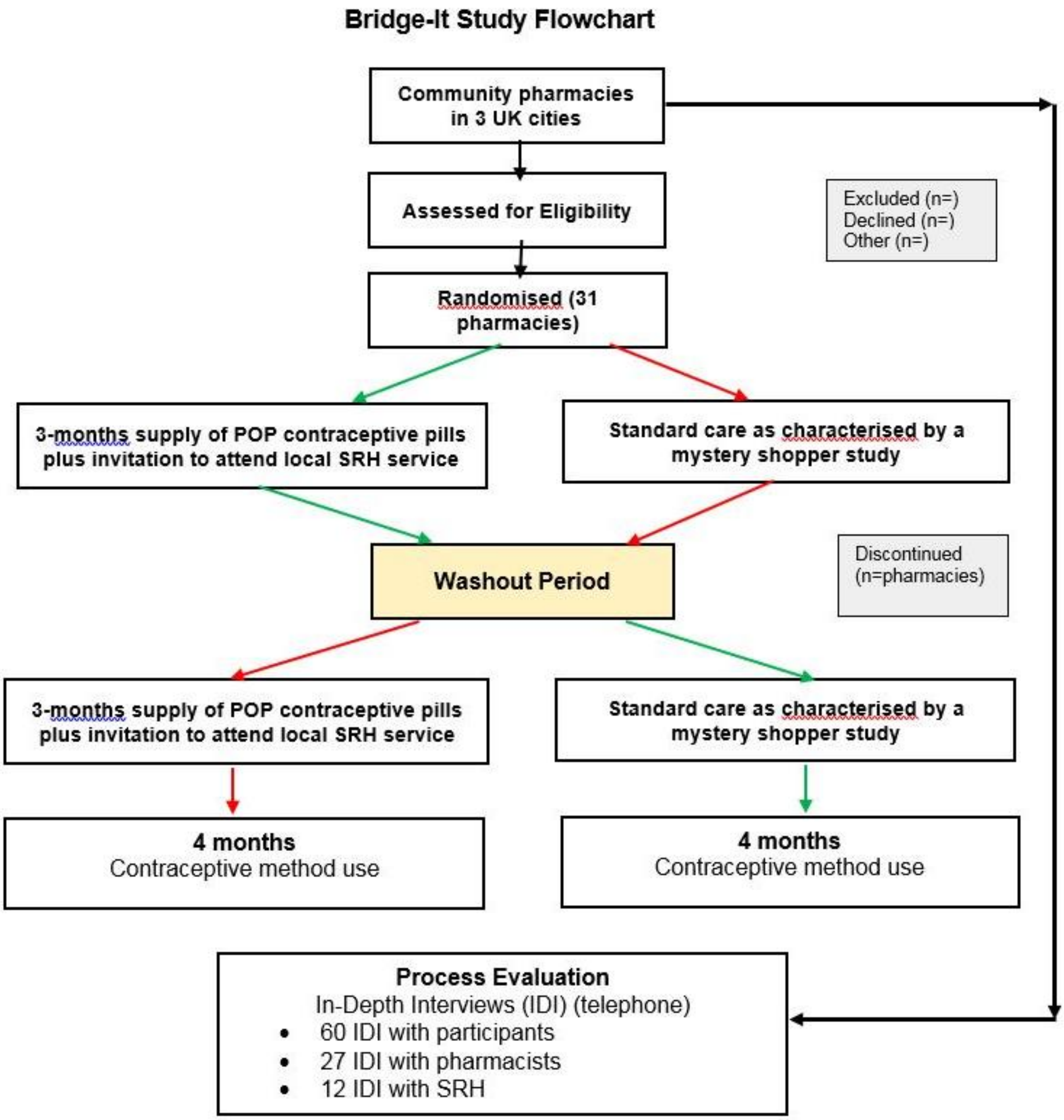

BMJ Open

A pragmatic cluster randomised cohort crossover trial to determine the effectiveness of bridging from emergency to regular contraception: The Bridge–It study protocol

Journal:	*BMJ Open*
Manuscript ID	bmjopen-2019-029978.R1
Article Type:	Protocol
Date Submitted by the Author:	09-Aug-2019

Complete List of Authors:	Cameron, Sharon; University of Edinburgh, Obstetrics and Gynaecology; NHS Lothian, Sexual and Reproductive Health<br>Baraitser, Paula; King's College London School of Medical Education<br>Glasier, Anna; University of Edinburgh<br>McDaid, Lisa; University of Glasgow, MRC/CSO Social and Public Health Sciences Unit<br>Norrie, John; Edinburgh Clinical Trials Unit, University of Edinburgh No. 9, Bioquarter<br>Radley, Andrew; NHS Tayside, Directorate of Public Health; University of Dundee Division of Cardiovascular and Diabetes Medicine, Liver Group<br>Stephenson, Judith; University College London, Instiute for Women's Health<br>Trussell, James; Princeton university, Office of Population Research<br>Battison, Claire; Edinburgh Clinical Trials Unit, University of Edinburgh No. 9, Bioquarter<br>Cameron, Sarah; University of Aberdeen, Health Services Research Unit<br>Cowle, Kathleen; Boots UK Ltd<br>Forrest, Mark; University of Aberdeen, Health Services Research Unit<br>Gilson, Richard; University College London, Institute for Global Health<br>Goulao, Beatriz; University of Aberdeen, Health Services Research Unit<br>Johnstone, Anne; University of Edinburgh, Obstetrics and Gynaecology<br>McDonald, Alison; University of Aberdeen, Health Services Research Unit<br>Morelli, Alessandra; King's College London School of Medical Education<br>Patterson, Susan; University of Glasgow, MRC/CSO Social and Public Health Sciences Unit<br>Sally, Deirdre; University College London, Instiute for Women's Health<br>Stewart, Nicola; University College London, Instiute for Women's Health

**Primary Subject Heading**:	Sexual health
Secondary Subject Heading:	Public health
Keywords:	contraception, emergency contraception, pharmacy, unintended pregnancy, levonorgestrel, progestogen only pill

SCHOLARONE™
Manuscripts

A pragmatic cluster randomised cohort crossover trial to determine the effectiveness of bridging from emergency to regular contraception: The Bridge–It study protocol

Bridge-It Study Group*

***Sharon T Cameron[1], Paula Baraitser [2], Anna Glasier [1], Lisa McDaid [3], John Norrie [4], Andrew Radley [5], Judith Stephenson [6], James Trussell [7†],Claire Battison [4], Sarah Cameron [8] , Kathleen Cowle [9], Mark Forrest [8], Richard Gilson [10], Beatriz Goulao [8], Anne Johnstone [1], Alison McDonald [8], Alessandra Morelli [11], Susan Patterson [3], Deirdre Sally [6], Nicola Stewart [6]**

[1]Corresponding author: Professor Sharon Cameron, Consultant Gynaecologist, Address: 2a Chalmers Street, Edinburgh, EH3 9ESTel: 00 44 131 536 2091 Email: sharon.cameron@ed.ac.uk

Word Count: 3767

Funder: HTA/NIHR
Funding Reference Number: HTA Project 15/113/01
Protocol version 5, 8 January 2019

In addition, the following disclaimer should be included in this paper:

The views expressed are those of the author(s) and not necessarily those of the NIHR, the NHS or the Department of Health

For peer review only - http://bmjopen.bmj.com/site/about/guidelines.xhtml

ABSTRACT:

Introduction: Oral emergency contraception (EC) can prevent unintended pregnancy but it is important to start a regular method of contraception. Women in the UK usually access EC from a pharmacy but then need a subsequent appointment with a GP or a sexual and reproductive health (SRH) service to access regular contraception. Unintended pregnancies can occur during this time.

Methods and analysis: Bridge-It is a pragmatic cluster randomised cohort crossover trial designed to determine whether pharmacist provision of a bridging supply of a progestogen only pill (POP) plus rapid access to a local SRH clinic, results in increased uptake of effective contraception and prevents more unintended pregnancies than provision of EC alone. Bridge-It involves 31 pharmacies in three UK regions (London, Lothian and Tayside) aiming to recruit 626 to 737 women. Pharmacies will give EC (levonorgestrel) according to normal practice and recruit women to both intervention and the control phases of the study. In the intervention phase, pharmacists will provide the POP (desogestrel) and offer rapid access to a SRH clinic. In the control phase, pharmacists will advise women to attend a contraceptive provider for contraception (standard care).

Women will be asked four months later about contraceptive use. Data linkage to abortion registries will provide abortion rates over 12 months. The sample size is calculated on the primary outcome of effective contraception use at four months (yes/no) with 90% power and a 5% level of significance. Abortion rates will be an exploratory secondary analysis. Process evaluation includes interviews with pharmacists, SRH clinicians and women. Cost-effectiveness analysis will use a healthcare system perspective and be expressed as incremental cost-effectiveness ratio.

Ethics and dissemination: Ethical approval was received from South East Scotland REC June 2017. Results will be published in peer-reviewed journals and conference presentations.

Trial registration number ISRCTN 70616901; pre results

Strengths and limitations:

- Examines the important outcome of abortion rates over 1 year as an exploratory secondary analysis.
- Applicable only to women receiving levonogrestrel EC followed by a desogestrel POP
- Not applicable to use of ulipristal acetate for EC, since hormonal methods of contraception such as the desogestrel POP may interact with efficacy of ulipristal acetate, if started within five days

For peer review only - http://bmjopen.bmj.com/site/about/guidelines.xhtml

INTRODUCTION

Unintended pregnancy is widely perceived as a major public health problem. Unintended pregnancy commonly ends in abortion and the UK has among the highest abortion rates in Europe [1]. In 2017 almost 200,000 pregnancies ended in induced abortion [2, 3]. Unintended pregnancy also ends in childbirth; around 10% of UK births are unintended and 25% mistimed [4]. Unintended pregnancy is costly to the NHS (estimated to cost over £1 billion annually) [5] and can be distressing for women. Unintended pregnancies are more common in young women from deprived backgrounds, contributing to widening health inequalities for both mother and baby, and their families [2,3]. Unintended childbirth can have both socioeconomic consequences for women and their families and mental health consequences [6].

Oral emergency contraception (EC) prevents pregnancy in individual women following unprotected sex or contraceptive accidents. EC is only effective if taken before ovulation as it works by inhibiting or delaying ovulation [7]. Since EC became available from pharmacies in the UK without the need for a prescription, there has been a change in the pattern of access such that women who seek EC now choose to obtain this from a pharmacy rather than a contraceptive provider such as a general practitioner (GP) or sexual and reproductive health (SRH) service [8]. Although trials have shown that this facilitates access to EC and increases use, they have failed to show that this reduces unintended pregnancy rates within the population [9].

There are two types of EC: the most widely used EC contains the progestogen levonorgestrel and should be taken within 72 hours of sex; the other EC contains the progesterone receptor modulator ulipristal acetate and should be taken within 120 hours of sex [10]. Neither formulation of EC prevents conceptions from subsequent acts of sex and the risk of pregnancy is increased up to threefold among women who have further unprotected sex in the same menstrual cycle after using EC than those who do not [10]. An effective method of contraception should therefore be started as soon as possible [10, 11]. However, the only contraceptives that can be obtained from any pharmacy without a prescription are condoms, which have high failure rates [12]. This means that women usually need to make an appointment with a contraceptive provider (GP or SRH) and may experience delays in accessing regular contraception or lose the motivation to access a regular method altogether, which in turn may result in unintended pregnancies. In addition, although pharmacists in the UK are supposed to advise women on where to obtain ongoing contraception after EC, in one study fewer than half of pharmacists did so [13]. It is possible that if pharmacists could supply a temporary (bridging) method of contraception to women along with EC, this would bridge the gap until women could get an appointment with a contraceptive provider for contraceptive advice and supplies. The progestogen only pill (POP) is an effective method of contraception with few contraindications [14] making it safer than the combined oral contraceptive pill for pharmacy provision. However, studies have shown that starting hormonal contraception containing a progestogen within five days of ulipristal acetate may reduce the efficacy of EC and so only EC containing levonorgestrel is suitable for use in conjunction with a bridging method of hormonal contraception in this way [15, 16].

For peer review only - http://bmjopen.bmj.com/site/about/guidelines.xhtml

Pilot

In a pilot study in Edinburgh of 168 women presenting for EC [17] 11 pharmacies were randomised to one of three groups to provide EC (levonorgestrel) and either (i) standard advice on where to obtain ongoing contraception or (ii) one month of a progestogen only pill (POP) or (iii) the offer of rapid access to a local SRH service. Participants were contacted by telephone six to eight weeks later to determine their current contraceptive use. Compared to standard care, the proportion of women using effective contraception was significantly greater in both the POP (56% vs. 16% p=0.001) and the rapid access groups (52% vs. 16% p=0.027). This suggests that a supply of one month of POP after EC or rapid access from a pharmacy to a SRH service might increase short-term uptake of effective contraception following EC. We now propose a large randomised trial to determine whether a pharmacy-based intervention designed to facilitate the uptake of effective contraception after EC increases use of effective contraceptive methods including the most effective long acting reversible contraceptive methods (LARC) such as the contraceptive implant and intrauterine contraception [12] at four months when compared with standard care. We will examine contraceptive uptake at four months as most POP preparations are packaged as a three month supply and so by four months the pharmacy provided supply will have ended.

Aim

The aim is to develop a simple and affordable intervention which facilitates the uptake of effective ongoing contraception among women obtaining EC from pharmacies thereby reducing unintended pregnancy. The primary objective is to determine whether offering women attending a pharmacy for EC, a three month bridging supply of POP plus the offer of rapid access to a local SRH service results in increased uptake of effective contraception. The study POP (desogestrel) is commonly used in the UK. In contrast to other POP s, the desogestrel POP reliably inhibits ovulation and has similar effectiveness to a combined hormonal oral contraceptive pill (COCP), yet fewer contraindications than a COCP [14,15]. This combined intervention (POP plus rapid access) offers both a highly safe temporary method of contraception and facilitates access to a specialist contraceptive service where all methods of contraception including the most effective LARC methods can be provided. If this intervention leads to increased uptake of effective contraception including LARC methods compared to standard care alone then we might expect that this would translate into fewer unintended pregnancies for women.

METHODS AND ANALYSIS

Study design and setting

A pragmatic cluster randomised cohort crossover trial with cost effectiveness including process, outcome and economic evaluation involving 31 pharmacies in three UK regions (15 in London (South and Central), 12 in Lothian (Edinburgh and region) and four in Tayside (Dundee and region).

Patient and Public Involvement

The members of the Patient and Public Involvement (PPI) Group at the SRH service in Edinburgh contributed to the design of this study. The study protocol and

For peer review only - http://bmjopen.bmj.com/site/about/guidelines.xhtml

documentation were reviewed and approved by the chair and members of the PPI group. The plain English summary was edited by a PPI member and improved as a result. There are three PPI members that participate and contribute to the Bridge-It Trial steering Committee meeting (TSC) that provides oversight of the study. PPI group will be involved in the dissemination of the study results.

Intervention

The planned intervention is a composite intervention. Each woman in the intervention phase will receive three months of POP (in a single package covering 3 months) and an invitation to attend a local participating SRH service to discuss and obtain effective contraception (including LARC methods). Three packets of POP, (75 mcg desogestrel; UK) containing 28 tablets each will be provided at no cost to women as a bridging method of contraception, providing them with three months of contraception during which time they can get an appointment with a contraceptive provider to obtain their preferred method of contraception. Locally approved Patient Group Directions (i.e. strict criteria to permit provision of specified medicines by non-prescribers) will permit participating pharmacists to dispense the POP to women recruited to the study. Pre-study training will be undertaken with participating pharmacists including identifying medical contraindications to POP, potential drug interactions medications and 'missed pill' guidance for POP. Pharmacists will advise women to start the POP the day following intake of EC [10] and provide women with a patient information booklet on the POP from the family planning association (www.fpa.org).

Pharmacists will encourage women to attend the participating local SRH service to obtain the contraceptive method of their choice. Participants (intervention phase) will be given a study card to alert staff at SRH services that they are in the Bridge-It study and should be seen as a drop in for contraception that same day. This card will also provide written information about the location and opening hours of the local participating SRH service. These SRH services are within a 5-mile radius of the participating pharmacies and provide all methods of contraception at no cost as is the norm in the NHS.

Standard Care

A mystery shopper exercise [13] will be undertaken in all 31 participating pharmacies to characterise 'standard care' (usually verbal advice to visit a clinic for contraception, with/without written and verbal information) in the control phase. The mystery shopper visits will be conducted when the pharmacy is not recruiting and just before the control phase starts. The mystery shoppers and the scenario used will be chosen by the Patient Public Involvement group. A simple scenario relating to request for EC will be used. Immediately after leaving the pharmacy the mystery shopper will complete a standard data collection proforma, recording any information given by the pharmacist about use of contraception after taking EC, including provision of written information.

Participants

We will recruit a total of 626 to 737 women presenting for EC. The final number will be determined based on the observed ratio of the between-period within-cluster correlation (BPC) and the within-period within-cluster correlation (WPC) – with the

For peer review only - http://bmjopen.bmj.com/site/about/guidelines.xhtml

larger sample size (near the 737 upper limit) required for values of BPC close to zero, and the smaller sample size (near the 626 lower limits) required for values of BPC close to WPC (18). Each pharmacy will be expected to recruit an average of 12-13 women to the intervention arm and 12-13 women to standard care. This allows for a 25% loss to follow up at four months (missing data on primary outcome).

Randomisation

Each pharmacy will be randomised to either the intervention phase for approximately 20 weeks followed by standard care phase for 20 weeks or vice-versa with a wash-out period of two weeks between the two phases. The order in which pharmacy allocation to each arm is undertaken (intervention or control first) will be randomised (Figure 1).The order of delivery of intervention or control for each pharmacy was randomised for this cluster crossover design from a randomisation file prepared by the study statistician in the Data Centre at the Centre for Healthcare Randomised Trials (CHaRT), University of Aberdeen, using SAS 6.4 for Windows. The method used for generating the random unpredictable mix of permuted blocks was a computer software algorithm that randomly allocated blocks of size 2, 4 and 6; blocking was used to ensure balanced group sizes.

This is a cluster cohort crossover design so it is the pharmacy that is the unit of randomisation and the 'crossover' means that we are just randomising the order that each pharmacy gives the intervention in. The 'cohort' label means that we expect different women to be recruited within each site in the two periods (intervention and control phases).

Recruitment

The pharmacist will assess medical eligibility of women presenting for EC for the study, provide EC according to normal practice and invite eligible women to participate. A detailed Patient Information Sheet (PIS) will be provided to all women and informed consent will be obtained by participating community pharmacists. The EC used in this study is levonorgestrel and will be given in the clinically indicated dose for the woman's weight (1.5 mg or 3 mg levonorgestrel) [10].

Inclusion and exclusion criteria are shown in Table 1. Women who give written consent will be recruited in the study. We recognise the importance of participant retention and will offer a voucher of £10 at recruitment [19].

Outcomes

A full list of study outcome measures is included in Table 2. Outcomes at four months will primarily be collected via telephone interviews or via web-based questionnaires. However, participants will also have the option to provide the same information by a postal questionnaire. The primary outcome is use of effective contraception at four months (intervention vs standard care).

Secondary outcomes are proportion of participants having an abortion within 12 months of EC use using record linkage from participants to national registries and cost effectiveness.

Process evaluation measures

A process evaluation will be conducted as part of the study to assess potential issues concerning intervention implementation, the causal mechanisms of impact, and the

For peer review only - http://bmjopen.bmj.com/site/about/guidelines.xhtml

contextual factors that could affect these. The process evaluation will comprise of quantitative and qualitative data measures, as detailed in Table 3.

Data Collection

Quantitative data

Participant flow: Participant flow through the study will be assessed and reported following the CONSORT flow chart.

Baseline

Participant demographics and reproductive history is collected at recruitment by a self-administered paper questionnaire given to them by the pharmacist. Demographic data will also be reported for the process evaluation, protocol adherence checklists and for recruitment screening forms (see Table 3).

Contraceptive use at 4 months

This will be based upon self-reported data from women at a telephone follow up interview with a research nurse at four months after obtaining EC. If participants prefer, the questions can be self-completed by a web-based questionnaire or paper questionnaire sent by post. Women will be asked what method of contraception they are using (if any), if they attended a GP or SRH service for this, if they used the POP (intervention phase only) and their pregnancy status. If pregnancy has occurred since EC then the validated London Measure of Unintended Pregnancy tool will be administered to measure intended-ness of the index pregnancy [20] (Table 2).

Abortion rates at 12 months

Information Services Division (ISD Scotland) and Department of Health (DOH England) will provide the number of abortions occurring during the 12 month follow up period in each arm by conducting linkage of the identifiers (collected at baseline) from study participants.

Validation

We will check with data from local SRH services to determine the numbers of participants from intervention and control phases who attend the local participating SRH service, and which method of contraception they received.

Qualitative data

Semi-structured, qualitative telephone interviews of a purposive sample of up to 60 women who received the intervention (approximately 22 in London, 30 in Edinburgh and 8 in Dundee) will be undertaken. Participants who consent to these telephone interviews will be contacted by the Process Evaluation Research Assistant. Interviews will explore experience of intervention acceptability in more depth and assess experiences of bridging from EC to regular contraception, and reasons for doing so or not (Table 3). Interviews will be conducted soon after the four month follow up.

Interviews with 27 pharmacists and 12 SRH service providers will explore their perceptions of barriers and facilitators to implementation and more broadly, their views on the intervention, the trial, and the target population. Interviews will be

For peer review only - http://bmjopen.bmj.com/site/about/guidelines.xhtml

conducted by the process evaluation research assistant soon after the intervention phase has completed.

For the process evaluation, data collection also includes: review of training and materials; observation of training; mapping of local contraceptive services within 10 miles of study sites and monitoring of contemporaneous events, such as relevant high coverage media stories using Google Alerts (Table 3).

Sample size calculation
The study is a pragmatic cluster randomised cohort crossover trial. Ideally the control and intervention phases should be of roughly equal duration and size, and the sample size is calculated assuming an equal cluster size in both control and intervention periods, and equal across sites (the expected site/period average). In practice, there is variability in EC demand across sites and over time: for example, demand is affected by peak holiday periods (recruitment decreases) and student term times (recruitment increases); and the ability of a pharmacy to translate that variable demand into study recruits depends on many factors, including changes to circumstances at individual pharmacies or loss of / change of pharmacists at multi-pharmacist stores.
Informed by our pilot study, we have assumed that effective contraception use in the control would be 30% and we were likely to achieve a 50% relative improvement to 45%. This means the sample size is in the range 626 to 737, assuming 25% of women do not provide four-month contraceptive use data, and an average cluster size of 12-13 in each period, and around 25 pharmacies taking part, with 90% power and a 5% level of significance. The uncertainty in the required sample size rests on the assumed between-period within-cluster correlation (BPC) and its relationship to the other component of variability, the within-period within-cluster correlation (WPC)[18]. The WPC is the usual correlation (known as the Intra Class Correlation–ICC in a standard parallel groups non-crossover cluster setting) of two individuals' outcomes within a cluster (in the same period). The BPC on the other hand is the correlation between two individuals' outcomes in the same cluster between the two periods. If the BPC is zero, there is no advantage in a crossover design over the standard parallel groups cluster design; if the BPC equals the WPC then the crossover is as efficient as an individually randomised design. We will finalise the sample size depending on the observed ratio of the BPC to WPC, and the observed rate of attrition, but still assuming the same control rate and treatment effect, once we have 4 month data on at least 500 participants.

Quantitative Analyses
There will be a single analysis at study end (there is no opportunity for any interim analyses given the crossover design) although an independent Data Monitoring Committee will monitor study progress and any safety issues. This will follow the intention to treat principle and will use a hierarchical model appropriate for the specific outcome. For the primary outcome this will be a mixed effects logistic regression, using the hierarchical model approach as recommended by Turner for a cluster crossover design [21]. We will pre-specify any individual level (or cluster level) covariates that we intend to adjust for, and the comprehensive Statistical Analysis Plan will specify the sensitivity type analyses that will explore how robust

For peer review only - http://bmjopen.bmj.com/site/about/guidelines.xhtml

the findings are to any missing data at the cluster level (probably unlikely) and the individual level (expected to be substantial for the patient reported outcomes at four months). As well as the usual assumption of missing at random, we will try to explore possible mechanisms for non-ignorable (informative missingness) at the individual level which may well be operating in this context. Subgroup analyses will explore the possible effect modification by LARC (most effective contraceptive methods) vs non-LARC vs no use of contraception.

Qualitative and mixed-methods process evaluation analyses

All process data will be analysed independently of the outcome data and, importantly, documented before the outcomes are known. Qualitative analysis of in -depth interviews will be recorded and transcribed verbatim. Transcription and analysis (proceeding case by case) will start with the first interview and be ongoing during the course of data collection, allowing for emergent themes to be identified and explored in future interviews. The transcripts will be read repeatedly and coded for analysis. Data management will be assisted by the software, QSR NVivo 10. Analysis will be undertaken using 'Framework Analysis' a method of proven validity and reliability where data are coded, indexed and charted systematically, then organised using a matrix or framework [22]. Constant comparison will be carried out to ensure that the analysis represents all perspectives and negative ('deviant') cases. The multi-source process evaluation will be synthesised to address the three key process evaluation questions: i) what was delivered, ii) how it was delivered, and iii) what role context may have had in shaping the delivery/outcomes

Economic evaluation: An economic evaluation will be undertaken comparing the intervention and control arms in a cost effectiveness analysis. A trial-based analysis will be followed by the construction of a decision model to extrapolate future costs and benefits beyond the completion of the trial. The overall perspective used will be that of the health system. Costs will include the pharmacist training to provide POP, direct and indirect costs of health service use, and the provision and dispensing of POP. We will compare the costs to the NHS in the intervention and control arms. To account for differences in the numbers of women in the two arms, we will compare the cost per woman in each arm. In the control arm, the costs are (i) cost of EC, (ii) cost of pharmacist provision of EC, (iii) cost of abortions. In the intervention arm, the costs are in addition to these (iv) the cost of the POP, (v) cost of pharmacist training to provide POP and (vi) cost of pharmacist provision of POP. The costs (i) and (ii) are the same in both groups and so the extra cost of the intervention will be the sum of (iv), (v) and (vi). The cost per women who has an abortion is the same in both groups except that we hypothesise that the abortion rate will be lower in the intervention group. We can then state the outcome as conventional incremental cost effectiveness ratio i.e. for every £100 spent on the intervention results in x fewer abortions for a savings of £Y. If Y is greater than 100 then the intervention is cost effective. We will examine the sensitivity of the outcomes to variations in the costs of iv, v, and vi.

Data Management and Clinical Trial Unit support

Data will be collected on a paper case report form and will be entered directly into the trial database. Data will be entered into a trial database by pharmacists, research

For peer review only - http://bmjopen.bmj.com/site/about/guidelines.xhtml

nurses or staff at the trial co-ordinating centre. The data management and statistical support (including responsibility of data and final dataset) for the study is provided by the UKCRC registered Clinical Trial Unit (CTU) the Centre for Healthcare Randomised Trials (CHaRT) at the University of Aberdeen while the trial management is provided by another UKCRC registered CTU, the Edinburgh Clinical Trials Unit (ECTU) at Edinburgh University.

Ethics and dissemination

The Bridge-It trial involves procedures and medications which are well established in current NHS clinical practice and use. Adverse events may occur during or after the use of EC or POP and are well documented in the POP patient information leaflet. Serious adverse events will be recorded at the four month follow up interview and reported to the study sponsor. The study will be conducted in accordance with the principles of Good Clinical Practice (GCP).

A favorable ethical opinion has been obtained from the South East Scotland REC in June 2017. Approvals have been obtained from NHS Research Scotland (NRS) and Health Research Authority (HRA) England prior to commencement of the study. Annual progress reports and a final report at the conclusion of the trial will be submitted to REC within the timelines defined in the regulations. Protocol modifications are communicated by the ECTU to study sites via email and electronic newsletters.

The Bridge-It study website will include trial materials, trial progress, and summaries of key findings. In addition, public engagement and dissemination will also be undertaken via our Patient Public Involvement group.

The results of the study will be published in the academic journals and all participants will be offered a lay summary of the main findings of the study. The findings will also be presented at national and international conferences and disseminated via social media.

Trial Status

As at 7th Feb 2019 the study had recruited 503 participants across 29 sites. Recruitment to the first period completed on 13th Jan 2019, with 391 participants recruited at 29 sites. Recruitment is scheduled to be completed by Jun 2019, with analyses of the 4-month primary outcome expected to be available by Oct 2019.

DISCUSSION

Unintended pregnancy is widely perceived as a major public health problem. The proposed intervention in the Bridge-It study provides both temporary contraception (the POP) and facilitates access to effective contraception at a local SRH clinic. The cluster design was felt necessary for logistical reasons and confirmed in the qualitative work of our pilot study [17, 23] that an individually randomised trial would simply not recruit, as it was not feasible for pharmacists within a busy pharmacy to take additional time to randomise each individual. The crossover nature of the cluster design was chosen for efficiency, and by having a different set of women recruited at a pharmacy in the two different periods we avoided contamination by the participant. The purpose of the washout out period (was to minimise intervention effect carrying over from one period to another, as part of any contamination effect mediated by the pharmacist. With the cluster crossover design,

For peer review only - http://bmjopen.bmj.com/site/about/guidelines.xhtml

each cluster will act as its own control and fewer pharmacies are required than with a parallel cluster design.

If our proposed intervention works, then this could prevent more unintended pregnancies for more women. If the intervention is cost effective then it could have cost savings for the NHS.

REFERENCES.

1. Sedgh G, Singh S, Shah IH et al. Induced abortion: incidence and trends worldwide from 1995 to 2008. *Lancet*. 2012Feb 18;379(9816):625-32.

2.Department of Health. Abortion Statistics, England and Wales: 2017. https://www.gov.uk/government/publications/report-on-abortion-statistics-in-england-and-wales-for-2014 (access date 11 Jan 2019)

3. Information Services Division. Termination of Pregnancy Statistics 2017 http://www.isdscotland.org/Health-Topics/Sexual-Health/Abortions/ (access date 11 Jan 2019)

4. Wellings K, Jones KG, Mercer CH et al. The prevalence of unplanned pregnancy and associated factors in Britain: findings from the third National Survey of Sexual Attitudes and Lifestyles (Natsal-3). *Lancet* 2013;382:1807-16

5. Thomas C, Cameron ST. Can we reduce costs and prevent more unintended pregnancies? A cost of illness study illustrating the cost-saving potential of a more effective method of EHC. *BMJ open* 2013;3:e003815.doi:10.1136

6. SmithBattle L, Leonard V. Inequities compounded: explaining variations in the transition to adulthood for teen mothers' offspring. *J Fam Nurs*. 2012;18(3):409-31.

7. Cameron ST, Li H R**,** Gemzell –Danielsson K. Current controversies with Emergency Contraception: a review. *BJOG* 2017(13):1948-1956.

8. Marston C, Meltzer H, Majeed A. Impact on contraceptive practice of making emergency hormonal contraception available over the counter in Great Britain: repeated cross sectional surveys.*BMJ*. 2005Jul 30;331(7511):271.

9. Polis CB, Grimes DA, Schaffer K, et al . Advance provision of emergency contraception for pregnancy prevention. Cochrane Database of Systematic Reviews 2007,Issue2.Art. No: CD005497. DOI:0.1002/14651858.CD005497.pub2.

10. Faculty of Sexual and Reproductive Healthcare, UK. Emergency contraception. 2017. www.fsrh.org (access date 11 January 2019)

11. Cheng L, Che Y, Gülmezoglu AM. Interventions for emergency contraception. *Cochrane Database of Systematic Reviews* 2012;Issue 8. Art. No.: CD001324. DOI: 10.1002/14651858.CD001324.pub4.

12. Trussell J. Contraceptive efficacy. Global Library of Womens

For peer review only - http://bmjopen.bmj.com/site/about/guidelines.xhtml

Medicinehttp://www.glowm.com/section_view/heading/Contraceptive%20Efficacy/item/374 (access date 29 Jan 2019)

13 .Glasier A, Manners RJ, Loudon JC, et al. Community pharmacists providing emergency contraception give little advice about future contraceptive use: a mystery shopper study. *Contraception* 2010;82: 538–42.

14. The Faculty of Sexual and Reproductive Healthcare (FSRH). UK medical Eligibility criteria for Contraceptive Use. http://www.fsrh.org/standards-and-guidance/uk-medical-eligibility-criteria-for-contraceptive-use/ (access date 11 Jan 2019)

15. Brache V, Cochon L, Duijkers IJ, et al. A prospective, randomized, pharmacodynamic study of quick-starting a desogestrel progestin-only pill following ulipristal acetate for emergency contraception. *Hum Reprod* 2015;30(12):2785-2793

16. Cameron ST, Berger C, Michie L,et al. The effects on ovarian activity of 'quickstarting' a combined oral contraceptive pill after ulipristal acetate : prospective, randomised, double-blind parallel-arm, placebo-controlled study. *Hum Reprod* 2015;30(7):1566-1572.

17. Michie L, Cameron ST, Glasier A et al .Pharmacy based interventions for initiating effective contraception following the use of emergency contraception: a pilot study. *Contraception* 2014 ;90, 447-53

18. Arnup SJ, McKenzie JE, Hemming K, et al. Understanding the cluster crossover design: a graphical illustration of the components of variation and a sample size tutorial. *Trials.* 2017;18; 381.

19. Brueton VC, Tierney J, Stenning S, et al. Strategies to improve retention in randomised trials. Cochrane Database Syst Rev. 2013 Dec 3; 12:MR000032. doi: 10.1002/14651858.MR000032.pub2. 21

20. Cameron ST ,Glasier A. Identifying women in need of further discussion about the decision to have an abortion and eventual outcome. *Contraception*. 2013;88(1): 128-32

21. Turner RM, Omar RZ, Thompson SG. Modelling multivariate outcomes in hierarchical data, with application to cluster randomised trials. *Biom J*. 2006 Jun;48(3):333-45.

22. Ritchie J, Spencer L. Qualitative research practice: a guide for social science students and researchers. Sage: London, 2003.

23. Michie L, Cameron ST, Glasier A et al. Providing bridging contraception after emergency contraception from the pharmacy: a qualitative study. Public Health Public Health 2016; 135: 97-103.

For peer review only - http://bmjopen.bmj.com/site/about/guidelines.xhtml

Authors' contributions: STC, AG, JN, LMc D, AR, PB, JS, JT developed the original protocol. SC, CB, MF, RG, AMcD, BG, AJ, AM, SP,DS,NS, KC contributed to later stages of study design. STC wrote the draft of the protocol with significant input from all authors at all stages. All authors contributed, read and approved the final manuscript.

Author affiliations:

1. Chalmers Centre, NHS Lothian, Edinburgh EH3 9ES and University of Edinburgh
2. King's College Hospital NHS Foundation Trust, London, SE5 9RS
3. MRC/CSO Social & Public Health Sciences Unit, University of Glasgow, Glasgow G2 3AX
4. Edinburgh Clinical Trial Unit, Usher Institute, University of Edinburgh, Edinburgh EH16 4UX
5. King's Cross Hospital, NHS Tayside, Dundee DD3 8EA
6. University College London, EGA Institute for Women's Health, London WC1E 6AU
7. †Deceased December 2018. Princeton University USA.
8. Health Service Research Unit, University of Aberdeen, Aberdeen, AB25 2ZD
9. Boots UK, North Hub Office, Falkirk FK1 1ES
10. University College London, Institute for Global Health, London WC1E 6JB
11. King's College London, SE5 9RJ

Acknowledgements: Thank you to the members of the Patient and Public Involvement Group at the SRH service in Edinburgh who contributed to the design of this study. Thanks to the women who have participated in this study and to the community pharmacists, research staff and health care professionals at the local SRH services who have assisted with the implementation of this study. Thanks to Laura Flett for trial management support.

The Bridge-It Study Steering Committee provides oversight for this trial on behalf of the sponsor (University of Edinburgh and NHS Lothian) and the funder. The members are: Professor Peter Brocklehurst (Chair), Birmingham Clinical Trials Unit; Dr Lucy Michie, Sandyford Glasgow; Professor Kaye Wellings, London School of Hygiene and Tropical Medicine; Joanna Loudon, PPI member, Edinburgh; Kirsten Stuart PPI member Edinburgh; Emily Whittaker PPI member Edinburgh.

The Data Monitoring Committee is an independent multidisciplinary group consisting of clinicians and statisticians. The members are: Professor Claire Anderson (Chair), University of Nottingham; Professor Elizabeth Allen, London School of Hygiene and Tropical Medicine; Professor Caroline Moreau, Johns Hopkins Bloomberg School of Public Health, USA. A copy of the DMC charter is held in Edinburgh Clinical Trials Unit.

For peer review only - http://bmjopen.bmj.com/site/about/guidelines.xhtml

The study has co-sponsorship between The University Court of the University of Edinburgh and Lothian Health Board. The sponsors representative is accord@nhslothian.scot.nhs.uk

The paper presents independent research funded by the National Institute for Health Research (NIHR). The views expressed are those of the authors and not necessarily those of the NHS, the NIHR or the Department of Health.

Funding statement: The Bridge-It study is funded by the National Institute for Health Research's Health Technology Assessment Programme. HTA Project:15/113/01

Competing interests statement.

AG is a member of HRA Pharma scientific advisory board.

PB is a Clinical Director of the not-for profit community interest company SH:24 that provides online sexual health services in partnership with the NHS.

AR Research grants, educational grants and consultancy with Gilead
Research grants from Roche and BMS
Educational grants from Abbvie

JN is Deputy Chair of the NIHR/HTA General Board Committee. NIHR/HTA funded this research.

For peer review only

Table 1. Inclusion and exclusion criteria

**Inclusion Criteria**<br>• Intake of EC (levonorgestrel)<br>• Capacity to give informed consent to participate in the trial which includes adherence to trial requirements<br>• Age 16 years or over<br>• Willing to give contact details and be contacted at four months by phone or text or e-mail or post<br>• Willing to give identifying data sufficient to allow data linkage with NHS registries
**Exclusion Criteria**<br>• Contraindications to the POP<br>• On medication that interacts adversely with POP<br>• Already using a hormonal method of contraception<br>• Require interpreting services<br>• If pharmacist has concerns about non-consensual sex

For peer review only

For peer review only - http://bmjopen.bmj.com/site/about/guidelines.xhtml

Table 2. Study outcomes

		Data Source
Main outcome	Use of effective contraceptive method (hormonal or intrauterine) in intervention vs control at 4 months	Self reported (telephone or self completed survey) at 4 months
Secondary Outcomes	Numbers undergoing an abortion within 12 months intervention vs control	National abortion registries
	Economic evaluation	Incremental cost effectiveness ratio for pregnancies prevented

For peer review only

For peer review only - http://bmjopen.bmj.com/site/about/guidelines.xhtml

Table 3: Process evaluation methods and data

**Theory**
Theory of change model
**Study Team**
Pharmacy recruitment forms (study team members involved in recruitment will routinely record decision making contributing to pharmacy selection, including: number of contacts made; responses from potential pharmacists; rationales for inclusion/exclusion; and reasons for refusal)
**Pharmacists**
Participant observation of training & review of training and intervention materials<br>Recruitment monitoring forms (n=100% of pharmacists) & protocol adherence checklists (n=100% of pharmacists)<br>Follow-up semi-structured telephone interviews with pharmacists (n=27; one with each pharmacy involved).
**SRH Providers**
Semi-structured telephone interviews with SRH providers (n=12; with Service Manager, mix staff at 2x services in London; 1x service in Edinburgh; 1x service in Dundee).
**Participants**
Telephone questionnaire administered by Research Nurse at 4 months post-intervention (n=100% participants)<br>Semi-structured telephone interviews at 4 months post-intervention (n=60; 22 in London, 30 in Edinburgh and 8 in Dundee)
**Context**
Audit of local contraceptive services within 10 miles of study sites in London, Edinburgh and Dundee<br>Monitoring of contemporaneous events, such as relevant high coverage media stories using Google Alerts

For peer review only - http://bmjopen.bmj.com/site/about/guidelines.xhtml

Figure Legend

Figure 1. Bridge-it flowchart

POP= progestogen only pill
IDI= in depth interviews
SRH= sexual and reproductive health

For peer review only

For peer review only - http://bmjopen.bmj.com/site/about/guidelines.xhtml

Figure 1: Bridge-It study flowchart

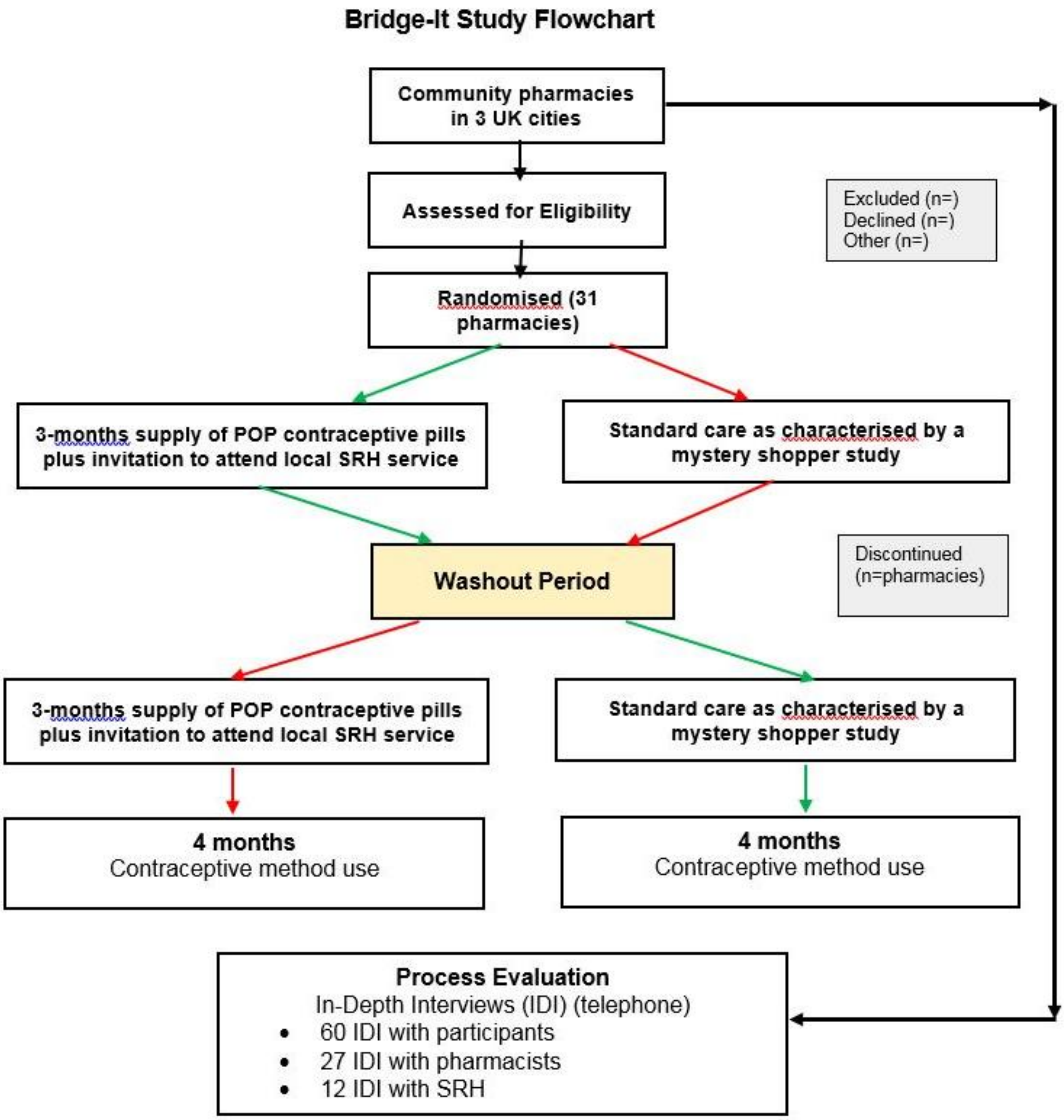

For peer review only - http://bmjopen.bmj.com/site/about/guidelines.xhtml

SPIRIT

STANDARD PROTOCOL ITEMS: RECOMMENDATIONS FOR INTERVENTIONAL TRIALS

SPIRIT 2013 Checklist: Recommended items to address in a clinical trial protocol and related documents*

Section/item	Item No	Description: Bridge it study	Addressed on page number
**Administrative information**			
Title	1	Descriptive title identifying the study design, population, interventions, and, if applicable, trial acronym	__________1____
Trial registration	2a	Trial identifier and registry name. If not yet registered, name of intended registry	___________1___
	2b	All items from the World Health Organization Trial Registration Data Set	____________N/A_
Protocol version	3	Date and version identifier	_____1_________
Funding	4	Sources and types of financial, material, and other support	______________1
Roles and responsibilities	5a	Names, affiliations, and roles of protocol contributors	________1,13_____
	5b	Name and contact information for the trial sponsor	_______13________
	5c	Role of study sponsor and funders, if any, in study design; collection, management, analysis, and interpretation of data; writing of the report; and the decision to submit the report for publication, including whether they will have ultimate authority over any of these activities	___________13___
	5d	Composition, roles, and responsibilities of the coordinating centre, steering committee, endpoint adjudication committee, data management team, and other individuals or groups overseeing the trial, if applicable (see Item 21a for data monitoring committee)	_____________13_

For peer review only

Introduction

Background and rationale	6a	Description of research question and justification for undertaking the trial, including summary of relevant studies (published and unpublished) examining benefits and harms for each intervention	______________3, 4
	6b	Explanation for choice of comparators	_3_____________
Objectives	7	Specific objectives or hypotheses	__4____________
Trial design	8	Description of trial design including type of trial (eg, parallel group, crossover, factorial, single group), allocation ratio, and framework (eg, superiority, equivalence, noninferiority, exploratory)	2,4 ______________

Methods: Participants, interventions, and outcomes

Study setting	9	Description of study settings (eg, community clinic, academic hospital) and list of countries where data will be collected. Reference to where list of study sites can be obtained	_4_____________
Eligibility criteria	10	Inclusion and exclusion criteria for participants. If applicable, eligibility criteria for study centres and individuals who will perform the interventions (eg, surgeons, psychotherapists)	Table1__________ __
Interventions	11a	Interventions for each group with sufficient detail to allow replication, including how and when they will be administered	___5___________
	11b	Criteria for discontinuing or modifying allocated interventions for a given trial participant (eg, drug dose change in response to harms, participant request, or improving/worsening disease)	__N/A___________ _
	11c	Strategies to improve adherence to intervention protocols, and any procedures for monitoring adherence (eg, drug tablet return, laboratory tests)	_________6_____
	11d	Relevant concomitant care and interventions that are permitted or prohibited during the trial	__________N/A___ _
Outcomes	12	Primary, secondary, and other outcomes, including the specific measurement variable (eg, systolic blood pressure), analysis metric (eg, change from baseline, final value, time to event), method of aggregation (eg, median, proportion), and time point for each outcome. Explanation of the clinical relevance of chosen efficacy and harm outcomes is strongly recommended	___6___________

For peer review only - http://bmjopen.bmj.com/site/about/guidelines.xhtml

Participant timeline	13	Time schedule of enrolment, interventions (including any run-ins and washouts), assessments, and visits for participants. A schematic diagram is highly recommended (see Figure)	_____Fig1________
Sample size	14	Estimated number of participants needed to achieve study objectives and how it was determined, including clinical and statistical assumptions supporting any sample size calculations	_________8____
Recruitment	15	Strategies for achieving adequate participant enrolment to reach target sample size	_________6____

Methods: Assignment of interventions (for controlled trials)

Allocation:

Sequence generation	16a	Method of generating the allocation sequence (eg, computer-generated random numbers), and list of any factors for stratification. To reduce predictability of a random sequence, details of any planned restriction (eg, blocking) should be provided in a separate document that is unavailable to those who enrol participants or assign interventions	____6_________
Allocation concealment mechanism	16b	Mechanism of implementing the allocation sequence (eg, central telephone; sequentially numbered, opaque, sealed envelopes), describing any steps to conceal the sequence until interventions are assigned	_________6____
Implementation	16c	Who will generate the allocation sequence, who will enrol participants, and who will assign participants to interventions	____6 _N/A as cluster randomised________
Blinding (masking)	17a	Who will be blinded after assignment to interventions (eg, trial participants, care providers, outcome assessors, data analysts), and how	__N/A___________
	17b	If blinded, circumstances under which unblinding is permissible, and procedure for revealing a participant's allocated intervention during the trial	_____N/A________

Methods: Data collection, management, and analysis

Data collection methods	18a	Plans for assessment and collection of outcome, baseline, and other trial data, including any related processes to promote data quality (eg, duplicate measurements, training of assessors) and a description of study instruments (eg, questionnaires, laboratory tests) along with their reliability and validity, if known. Reference to where data collection forms can be found, if not in the protocol	_________7,9_____
	18b	Plans to promote participant retention and complete follow-up, including list of any outcome data to be collected for participants who discontinue or deviate from intervention protocols	___________6__
Data management	19	Plans for data entry, coding, security, and storage, including any related processes to promote data quality (eg, double data entry; range checks for data values). Reference to where details of data management procedures can be found, if not in the protocol	____________9_
Statistical methods	20a	Statistical methods for analysing primary and secondary outcomes. Reference to where other details of the statistical analysis plan can be found, if not in the protocol	__________8,9
	20b	Methods for any additional analyses (eg, subgroup and adjusted analyses)	_8,9____________
	20c	Definition of analysis population relating to protocol non-adherence (eg, as randomised analysis), and any statistical methods to handle missing data (eg, multiple imputation)	_8____________

Methods: Monitoring

Data monitoring	21a	Composition of data monitoring committee (DMC); summary of its role and reporting structure; statement of whether it is independent from the sponsor and competing interests; and reference to where further details about its charter can be found, if not in the protocol. Alternatively, an explanation of why a DMC is not needed	_13____________
	21b	Description of any interim analyses and stopping guidelines, including who will have access to these interim results and make the final decision to terminate the trial	____N/A_________
Harms	22	Plans for collecting, assessing, reporting, and managing solicited and spontaneously reported adverse events and other unintended effects of trial interventions or trial conduct	_________10____
Auditing	23	Frequency and procedures for auditing trial conduct, if any, and whether the process will be independent from investigators and the sponsor	__N/A___________

Ethics and dissemination

Research ethics approval	24	Plans for seeking research ethics committee/institutional review board (REC/IRB) approval	_______2______
Protocol amendments	25	Plans for communicating important protocol modifications (eg, changes to eligibility criteria, outcomes, analyses) to relevant parties (eg, investigators, REC/IRBs, trial participants, trial registries, journals, regulators)	________10_____
Consent or assent	26a	Who will obtain informed consent or assent from potential trial participants or authorised surrogates, and how (see Item 32)	_________6____
	26b	Additional consent provisions for collection and use of participant data and biological specimens in ancillary studies, if applicable	___N/A_________ _
Confidentiality	27	How personal information about potential and enrolled participants will be collected, shared, and maintained in order to protect confidentiality before, during, and after the trial	_____This is in ethical application-on bridge it website _____
Declaration of interests	28	Financial and other competing interests for principal investigators for the overall trial and each study site	14 Also BMJ open IJMEC forms________
Access to data	29	Statement of who will have access to the final trial dataset, and disclosure of contractual agreements that limit such access for investigators	__page 9
Ancillary and post-trial care	30	Provisions, if any, for ancillary and post-trial care, and for compensation to those who suffer harm from trial participation	__ PIS, and consent__uploaded as supplementary___ ______
Dissemination policy	31a	Plans for investigators and sponsor to communicate trial results to participants, healthcare professionals, the public, and other relevant groups (eg, via publication, reporting in results databases, or other data sharing arrangements), including any publication restrictions	__10___________

For peer review only - http://bmjopen.bmj.com/site/about/guidelines.xhtml

	31b	Authorship eligibility guidelines and any intended use of professional writers	____12___________
	31c	Plans, if any, for granting public access to the full protocol, participant-level dataset, and statistical code	_________N/A_____ _

Appendices

Informed consent materials	32	Model consent form and other related documentation given to participants and authorised surrogates	__uploaded as supplementary files<br>Participant consent form<br>Participant information sheet<br>Pharmacist/SRH provider information sheet<br>Pharmacist/SRH provided consent form<br>GDPR participant Information sheet
Biological specimens	33	Plans for collection, laboratory evaluation, and storage of biological specimens for genetic or molecular analysis in the current trial and for future use in ancillary studies, if applicable	N/a

*It is strongly recommended that this checklist be read in conjunction with the SPIRIT 2013 Explanation & Elaboration for important clarification on the items. Amendments to the protocol should be tracked and dated. The SPIRIT checklist is copyrighted by the SPIRIT Group under the Creative Commons "Attribution-NonCommercial-NoDerivs 3.0 Unported" license.

For peer review only

BMJ Open

A pragmatic cluster randomised cohort crossover trial to determine the effectiveness of bridging from emergency to regular contraception: The Bridge–It study protocol

Journal:	*BMJ Open*
Manuscript ID	bmjopen-2019-029978.R2
Article Type:	Protocol
Date Submitted by the Author:	06-Sep-2019
Complete List of Authors:	Cameron, Sharon; University of Edinburgh, Obstetrics and Gynaecology; NHS Lothian, Sexual and Reproductive Health<br>Baraitser, Paula; King's College London School of Medical Education<br>Glasier, Anna; University of Edinburgh<br>McDaid, Lisa; University of Glasgow, MRC/CSO Social and Public Health Sciences Unit<br>Norrie, John; Edinburgh Clinical Trials Unit, University of Edinburgh No. 9, Bioquarter<br>Radley, Andrew; NHS Tayside, Directorate of Public Health; University of Dundee Division of Cardiovascular and Diabetes Medicine, Liver Group<br>Stephenson, Judith; University College London, Instiute for Women's Health<br>Trussell, James; Princeton university, Office of Population Research<br>Battison, Claire; Edinburgh Clinical Trials Unit, University of Edinburgh No. 9, Bioquarter<br>Cameron, Sarah; University of Aberdeen, Health Services Research Unit<br>Cowle, Kathleen; Boots UK Ltd<br>Forrest, Mark; University of Aberdeen, Health Services Research Unit<br>Gilson, Richard; University College London, Institute for Global Health<br>Goulao, Beatriz; University of Aberdeen, Health Services Research Unit<br>Johnstone, Anne; University of Edinburgh, Obstetrics and Gynaecology<br>McDonald, Alison; University of Aberdeen, Health Services Research Unit<br>Morelli, Alessandra; King's College London School of Medical Education<br>Patterson, Susan; University of Glasgow, MRC/CSO Social and Public Health Sciences Unit<br>Sally, Deirdre; University College London, Instiute for Women's Health<br>Stewart, Nicola; University College London, Instiute for Women's Health
**Primary Subject Heading**:	Sexual health
Secondary Subject Heading:	Public health
Keywords:	contraception, emergency contraception, pharmacy, unintended pregnancy, levonorgestrel, progestogen only pill

SCHOLARONE™
Manuscripts

A pragmatic cluster randomised cohort crossover trial to determine the effectiveness of bridging from emergency to regular contraception: The Bridge–It study protocol

Bridge-It Study Group*

***Sharon T Cameron[1], Paula Baraitser [2], Anna Glasier [1], Lisa McDaid [3], John Norrie [4], Andrew Radley [5], Judith Stephenson [6], James Trussell [7†],Claire Battison [4], Sarah Cameron [8] , Kathleen Cowle [9], Mark Forrest [8], Richard Gilson [10], Beatriz Goulao [8], Anne Johnstone [1], Alison McDonald [8], Alessandra Morelli [11], Susan Patterson [3], Deirdre Sally [6], Nicola Stewart [6]**

[1]Corresponding author: Professor Sharon Cameron, Consultant Gynaecologist, Address: 2a Chalmers Street, Edinburgh, EH3 9ESTel: 00 44 131 536 2091 Email: sharon.cameron@ed.ac.uk

Word Count: 3767

Funder: HTA/NIHR
Funding Reference Number: HTA Project 15/113/01
Protocol version 5, 8 January 2019

In addition, the following disclaimer should be included in this paper:

The views expressed are those of the author(s) and not necessarily those of the NIHR, the NHS or the Department of Health

For peer review only

ABSTRACT:

Introduction: Oral emergency contraception (EC) can prevent unintended pregnancy but it is important to start a regular method of contraception. Women in the UK usually access EC from a pharmacy but then need a subsequent appointment with a GP or a sexual and reproductive health (SRH) service to access regular contraception. Unintended pregnancies can occur during this time.

Methods and analysis: Bridge-It is a pragmatic cluster randomised cohort crossover trial designed to determine whether pharmacist provision of a bridging supply of a progestogen only pill (POP) plus rapid access to a local SRH clinic, results in increased uptake of effective contraception and prevents more unintended pregnancies than provision of EC alone. Bridge-It involves 31 pharmacies in three UK regions (London, Lothian and Tayside) aiming to recruit 626 to 737 women. Pharmacies will give EC (levonorgestrel) according to normal practice and recruit women to both intervention and the control phases of the study. In the intervention phase, pharmacists will provide the POP (desogestrel) and offer rapid access to a SRH clinic. In the control phase, pharmacists will advise women to attend a contraceptive provider for contraception (standard care).

Women will be asked four months later about contraceptive use. Data linkage to abortion registries will provide abortion rates over 12 months. The sample size is calculated on the primary outcome of effective contraception use at four months (yes/no) with 90% power and a 5% level of significance. Abortion rates will be an exploratory secondary analysis. Process evaluation includes interviews with pharmacists, SRH clinicians and women. Cost-effectiveness analysis will use a healthcare system perspective and be expressed as incremental cost-effectiveness ratio.

Ethics and dissemination: Ethical approval was received from South East Scotland REC June 2017. Results will be published in peer-reviewed journals and conference presentations.

Trial registration number ISRCTN 70616901; pre results

Strengths and limitations:

- Examines the important outcome of abortion rates over 1 year as an exploratory secondary analysis.
- Applicable only to women receiving levonogrestrel EC followed by a desogestrel POP
- Not applicable to use of ulipristal acetate for EC, since hormonal methods of contraception such as the desogestrel POP may interact with efficacy of ulipristal acetate, if started within five days

For peer review only - http://bmjopen.bmj.com/site/about/guidelines.xhtml

INTRODUCTION

Unintended pregnancy is widely perceived as a major public health problem. Unintended pregnancy commonly ends in abortion and the UK has among the highest abortion rates in Europe [1]. In 2017 almost 200,000 pregnancies ended in induced abortion [2, 3]. Unintended pregnancy also ends in childbirth; around 10% of UK births are unintended and 25% mistimed [4]. Unintended pregnancy is costly to the NHS (estimated to cost over £1 billion annually) [5] and can be distressing for women. Unintended pregnancies are more common in young women from deprived backgrounds, contributing to widening health inequalities for both mother and baby, and their families [2,3]. Unintended childbirth can have both socioeconomic consequences for women and their families and mental health consequences [6].

Oral emergency contraception (EC) prevents pregnancy in individual women following unprotected sex or contraceptive accidents. EC is only effective if taken before ovulation as it works by inhibiting or delaying ovulation [7]. Since EC became available from pharmacies in the UK without the need for a prescription, there has been a change in the pattern of access such that women who seek EC now choose to obtain this from a pharmacy rather than a contraceptive provider such as a general practitioner (GP) or sexual and reproductive health (SRH) service [8]. Although trials have shown that this facilitates access to EC and increases use, they have failed to show that this reduces unintended pregnancy rates within the population [9].

There are two types of EC: the most widely used EC contains the progestogen levonorgestrel and should be taken within 72 hours of sex; the other EC contains the progesterone receptor modulator ulipristal acetate and should be taken within 120 hours of sex [10]. Neither formulation of EC prevents conceptions from subsequent acts of sex and the risk of pregnancy is increased up to threefold among women who have further unprotected sex in the same menstrual cycle after using EC than those who do not [10]. An effective method of contraception should therefore be started as soon as possible [10, 11]. However, the only contraceptives that can be obtained from any pharmacy without a prescription are condoms, which have high failure rates [12]. This means that women usually need to make an appointment with a contraceptive provider (GP or SRH) and may experience delays in accessing regular contraception or lose the motivation to access a regular method altogether, which in turn may result in unintended pregnancies. In addition, although pharmacists in the UK are supposed to advise women on where to obtain ongoing contraception after EC, in one study fewer than half of pharmacists did so [13]. It is possible that if pharmacists could supply a temporary (bridging) method of contraception to women along with EC, this would bridge the gap until women could get an appointment with a contraceptive provider for contraceptive advice and supplies. The progestogen only pill (POP) is an effective method of contraception with few contraindications [14] making it safer than the combined oral contraceptive pill for pharmacy provision. However, studies have shown that starting hormonal contraception containing a progestogen within five days of ulipristal acetate may reduce the efficacy of EC and so only EC containing levonorgestrel is suitable for use in conjunction with a bridging method of hormonal contraception in this way [15, 16].

For peer review only - http://bmjopen.bmj.com/site/about/guidelines.xhtml

Pilot

In a pilot study in Edinburgh of 168 women presenting for EC [17] 11 pharmacies were randomised to one of three groups to provide EC (levonorgestrel) and either (i) standard advice on where to obtain ongoing contraception or (ii) one month of a progestogen only pill (POP) or (iii) the offer of rapid access to a local SRH service. Participants were contacted by telephone six to eight weeks later to determine their current contraceptive use. Compared to standard care, the proportion of women using effective contraception was significantly greater in both the POP (56% vs. 16% p=0.001) and the rapid access groups (52% vs. 16% p=0.027). This suggests that a supply of one month of POP after EC or rapid access from a pharmacy to a SRH service might increase short-term uptake of effective contraception following EC. We now propose a large randomised trial to determine whether a pharmacy-based intervention designed to facilitate the uptake of effective contraception after EC increases use of effective contraceptive methods including the most effective long acting reversible contraceptive methods (LARC) such as the contraceptive implant and intrauterine contraception [12] at four months when compared with standard care. We will examine contraceptive uptake at four months as most POP preparations are packaged as a three month supply and so by four months the pharmacy provided supply will have ended.

Aim

The aim is to develop a simple and affordable intervention which facilitates the uptake of effective ongoing contraception among women obtaining EC from pharmacies thereby reducing unintended pregnancy. The primary objective is to determine whether offering women attending a pharmacy for EC, a three month bridging supply of POP plus the offer of rapid access to a local SRH service results in increased uptake of effective contraception. The study POP (desogestrel) is commonly used in the UK. In contrast to other POP s, the desogestrel POP reliably inhibits ovulation and has similar effectiveness to a combined hormonal oral contraceptive pill (COCP), yet fewer contraindications than a COCP [14,15]. This combined intervention (POP plus rapid access) offers both a highly safe temporary method of contraception and facilitates access to a specialist contraceptive service where all methods of contraception including the most effective LARC methods can be provided. If this intervention leads to increased uptake of effective contraception including LARC methods compared to standard care alone then we might expect that this would translate into fewer unintended pregnancies for women.

METHODS AND ANALYSIS

Study design and setting

A pragmatic cluster randomised cohort crossover trial with cost effectiveness including process, outcome and economic evaluation involving 31 pharmacies in three UK regions (15 in London (South and Central), 12 in Lothian (Edinburgh and region) and four in Tayside (Dundee and region).

Patient and Public Involvement

The members of the Patient and Public Involvement (PPI) Group at the SRH service in Edinburgh contributed to the design of this study. The study protocol and

For peer review only - http://bmjopen.bmj.com/site/about/guidelines.xhtml

documentation were reviewed and approved by the chair and members of the PPI group. The plain English summary was edited by a PPI member and improved as a result. There are three PPI members that participate and contribute to the Bridge-It Trial steering Committee meeting (TSC) that provides oversight of the study. PPI group will be involved in the dissemination of the study results.

Intervention

The planned intervention is a composite intervention. Each woman in the intervention phase will receive three months of POP (in a single package covering 3 months) and an invitation to attend a local participating SRH service to discuss and obtain effective contraception (including LARC methods). Three packets of POP, (75 mcg desogestrel; UK) containing 28 tablets each will be provided at no cost to women as a bridging method of contraception, providing them with three months of contraception during which time they can get an appointment with a contraceptive provider to obtain their preferred method of contraception. Locally approved Patient Group Directions (i.e. strict criteria to permit provision of specified medicines by non-prescribers) will permit participating pharmacists to dispense the POP to women recruited to the study. Pre-study training will be undertaken with participating pharmacists including identifying medical contraindications to POP, potential drug interactions medications and 'missed pill' guidance for POP. Pharmacists will advise women to start the POP the day following intake of EC [10] and provide women with a patient information booklet on the POP from the family planning association (www.fpa.org).

Pharmacists will encourage women to attend the participating local SRH service to obtain the contraceptive method of their choice. Participants (intervention phase) will be given a study card to alert staff at SRH services that they are in the Bridge-It study and should be seen as a drop in for contraception that same day. This card will also provide written information about the location and opening hours of the local participating SRH service. These SRH services are within a 5-mile radius of the participating pharmacies and provide all methods of contraception at no cost as is the norm in the NHS.

Standard Care

A mystery shopper exercise [13] will be undertaken in all 31 participating pharmacies to characterise 'standard care' (usually verbal advice to visit a clinic for contraception, with/without written and verbal information) in the control phase. The mystery shopper visits will be conducted when the pharmacy is not recruiting and just before the control phase starts. The mystery shoppers and the scenario used will be chosen by the Patient Public Involvement group. A simple scenario relating to request for EC will be used. Immediately after leaving the pharmacy the mystery shopper will complete a standard data collection proforma, recording any information given by the pharmacist about use of contraception after taking EC, including provision of written information.

Participants

We will recruit a total of 626 to 737 women presenting for EC. The final number will be determined based on the observed ratio of the between-period within-cluster correlation (BPC) and the within-period within-cluster correlation (WPC) – with the

For peer review only - http://bmjopen.bmj.com/site/about/guidelines.xhtml

larger sample size (near the 737 upper limit) required for values of BPC close to zero, and the smaller sample size (near the 626 lower limits) required for values of BPC close to WPC [18]. Each pharmacy will be expected to recruit an average of 12-13 women to the intervention arm and 12-13 women to standard care. This allows for a 25% loss to follow up at four months (missing data on primary outcome).

Randomisation

Each pharmacy will be randomised to either the intervention phase for approximately 20 weeks followed by standard care phase for 20 weeks or vice-versa with a wash-out period of two weeks between the two phases. The order in which pharmacy allocation to each arm is undertaken (intervention or control first) will be randomised (Figure 1).The order of delivery of intervention or control for each pharmacy was randomised for this cluster crossover design from a randomisation file prepared by the study statistician in the Data Centre at the Centre for Healthcare Randomised Trials (CHaRT), University of Aberdeen, using SAS 6.4 for Windows. The method used for generating the random unpredictable mix of permuted blocks was a computer software algorithm that randomly allocated blocks of size 2, 4 and 6; blocking was used to ensure balanced group sizes.

This is a cluster cohort crossover design so it is the pharmacy that is the unit of randomisation and the 'crossover' means that we are just randomising the order that each pharmacy gives the intervention in. The 'cohort' label means that we expect different women to be recruited within each site in the two periods (intervention and control phases).

Recruitment

The pharmacist will assess medical eligibility of women presenting for EC for the study, provide EC according to normal practice and invite eligible women to participate. A detailed Patient Information Sheet (PIS) will be provided to all women and informed consent will be obtained by participating community pharmacists. The EC used in this study is levonorgestrel and will be given in the clinically indicated dose for the woman's weight (1.5 mg or 3 mg levonorgestrel) [10].

Inclusion and exclusion criteria are shown in Table 1. Women who give written consent will be recruited in the study. We recognise the importance of participant retention and will offer a voucher of £10 at recruitment [19].

Outcomes

A full list of study outcome measures is included in Table 2. Outcomes at four months will primarily be collected via telephone interviews or via web-based questionnaires. However, participants will also have the option to provide the same information by a postal questionnaire. The primary outcome is use of effective contraception at four months (intervention vs standard care).

Secondary outcomes are proportion of participants having an abortion within 12 months of EC use using record linkage from participants to national registries and cost effectiveness.

Process evaluation measures

A process evaluation will be conducted as part of the study to assess potential issues concerning intervention implementation, the causal mechanisms of impact, and the

For peer review only - http://bmjopen.bmj.com/site/about/guidelines.xhtml

contextual factors that could affect these. The process evaluation will comprise of quantitative and qualitative data measures, as detailed in Table 3.

Data Collection

Quantitative data

Participant flow: Participant flow through the study will be assessed and reported following the CONSORT flow chart.

Baseline

Participant demographics and reproductive history is collected at recruitment by a self-administered paper questionnaire given to them by the pharmacist. Demographic data will also be reported for the process evaluation, protocol adherence checklists and for recruitment screening forms (see Table 3).

Contraceptive use at 4 months

This will be based upon self-reported data from women at a telephone follow up interview with a research nurse at four months after obtaining EC. If participants prefer, the questions can be self-completed by a web-based questionnaire or paper questionnaire sent by post. Women will be asked what method of contraception they are using (if any), if they attended a GP or SRH service for this, if they used the POP (intervention phase only) and their pregnancy status. If pregnancy has occurred since EC then the validated London Measure of Unintended Pregnancy tool will be administered to measure intended-ness of the index pregnancy [20] (Table 2).

Abortion rates at 12 months

Information Services Division (ISD Scotland) and Department of Health (DOH England) will provide the number of abortions occurring during the 12 month follow up period in each arm by conducting linkage of the identifiers (collected at baseline) from study participants.

Validation

We will check with data from local SRH services to determine the numbers of participants from intervention and control phases who attend the local participating SRH service, and which method of contraception they received.

Qualitative data

Semi-structured, qualitative telephone interviews of a purposive sample of up to 60 women who received the intervention (approximately 22 in London, 30 in Edinburgh and 8 in Dundee) will be undertaken. Participants who consent to these telephone interviews will be contacted by the Process Evaluation Research Assistant. Interviews will explore experience of intervention acceptability in more depth and assess experiences of bridging from EC to regular contraception, and reasons for doing so or not (Table 3). Interviews will be conducted soon after the four month follow up.

Interviews with 27 pharmacists and 12 SRH service providers will explore their perceptions of barriers and facilitators to implementation and more broadly, their views on the intervention, the trial, and the target population. Interviews will be

For peer review only - http://bmjopen.bmj.com/site/about/guidelines.xhtml

conducted by the process evaluation research assistant soon after the intervention phase has completed.

For the process evaluation, data collection also includes: review of training and materials; observation of training; mapping of local contraceptive services within 10 miles of study sites and monitoring of contemporaneous events, such as relevant high coverage media stories using Google Alerts (Table 3).

Sample size calculation

The study is a pragmatic cluster randomised cohort crossover trial. Ideally the control and intervention phases should be of roughly equal duration and size, and the sample size is calculated assuming an equal cluster size in both control and intervention periods, and equal across sites (the expected site/period average). In practice, there is variability in EC demand across sites and over time: for example, demand is affected by peak holiday periods (recruitment decreases) and student term times (recruitment increases); and the ability of a pharmacy to translate that variable demand into study recruits depends on many factors, including changes to circumstances at individual pharmacies or loss of / change of pharmacists at multi-pharmacist stores.

Informed by our pilot study, we have assumed that effective contraception use in the control would be 30% and we were likely to achieve a 50% relative improvement to 45%. This means the sample size is in the range 626 to 737, assuming 25% of women do not provide four-month contraceptive use data, and an average cluster size of 12-13 in each period, and around 25 pharmacies taking part, with 90% power and a 5% level of significance. The uncertainty in the required sample size rests on the assumed between-period within-cluster correlation (BPC) and its relationship to the other component of variability, the within-period within-cluster correlation (WPC)[18]. The WPC is the usual correlation (known as the Intra Class Correlation–ICC in a standard parallel groups non-crossover cluster setting) of two individuals' outcomes within a cluster (in the same period). The BPC on the other hand is the correlation between two individuals' outcomes in the same cluster between the two periods. If the BPC is zero, there is no advantage in a crossover design over the standard parallel groups cluster design; if the BPC equals the WPC then the crossover is as efficient as an individually randomised design. We will finalise the sample size depending on the observed ratio of the BPC to WPC, and the observed rate of attrition, but still assuming the same control rate and treatment effect, once we have 4 month data on at least 500 participants.

Quantitative Analyses

There will be a single analysis at study end (there is no opportunity for any interim analyses given the crossover design) although an independent Data Monitoring Committee will monitor study progress and any safety issues. This will follow the intention to treat principle and will use a hierarchical model appropriate for the specific outcome. For the primary outcome this will be a mixed effects logistic regression, using the hierarchical model approach as recommended by Turner for a cluster crossover design [21]. We will pre-specify any individual level (or cluster level) covariates that we intend to adjust for, and the comprehensive Statistical Analysis Plan will specify the sensitivity type analyses that will explore how robust

For peer review only - http://bmjopen.bmj.com/site/about/guidelines.xhtml

the findings are to any missing data at the cluster level (probably unlikely) and the individual level (expected to be substantial for the patient reported outcomes at four months). As well as the usual assumption of missing at random, we will try to explore possible mechanisms for non-ignorable (informative missingness) at the individual level which may well be operating in this context. Subgroup analyses will explore the possible effect modification by LARC (most effective contraceptive methods) vs non-LARC vs no use of contraception.

Qualitative and mixed-methods process evaluation analyses

All process data will be analysed independently of the outcome data and, importantly, documented before the outcomes are known. Qualitative analysis of in-depth interviews will be recorded and transcribed verbatim. Transcription and analysis (proceeding case by case) will start with the first interview and be ongoing during the course of data collection, allowing for emergent themes to be identified and explored in future interviews. The transcripts will be read repeatedly and coded for analysis. Data management will be assisted by the software, QSR NVivo 10. Analysis will be undertaken using 'Framework Analysis' a method of proven validity and reliability where data are coded, indexed and charted systematically, then organised using a matrix or framework [22]. Constant comparison will be carried out to ensure that the analysis represents all perspectives and negative ('deviant') cases. The multi-source process evaluation will be synthesised to address the three key process evaluation questions: i) what was delivered, ii) how it was delivered, and iii) what role context may have had in shaping the delivery/outcomes

Economic evaluation: An economic evaluation will be undertaken comparing the intervention and control arms in a cost effectiveness analysis. A trial-based analysis will be followed by the construction of a decision model to extrapolate future costs and benefits beyond the completion of the trial. The overall perspective used will be that of the health system. Costs will include the pharmacist training to provide POP, direct and indirect costs of health service use, and the provision and dispensing of POP. We will compare the costs to the NHS in the intervention and control arms. To account for differences in the numbers of women in the two arms, we will compare the cost per woman in each arm. In the control arm, the costs are (i) cost of EC, (ii) cost of pharmacist provision of EC, (iii) cost of abortions. In the intervention arm, the costs are in addition to these (iv) the cost of the POP, (v) cost of pharmacist training to provide POP and (vi) cost of pharmacist provision of POP. The costs (i) and (ii) are the same in both groups and so the extra cost of the intervention will be the sum of (iv), (v) and (vi). The cost per women who has an abortion is the same in both groups except that we hypothesise that the abortion rate will be lower in the intervention group. We can then state the outcome as conventional incremental cost effectiveness ratio i.e. for every £100 spent on the intervention results in x fewer abortions for a savings of £Y. If Y is greater than 100 then the intervention is cost effective. We will examine the sensitivity of the outcomes to variations in the costs of iv, v, and vi.

Data Management and Clinical Trial Unit support

Data will be collected on a paper case report form and will be entered directly into the trial database. Data will be entered into a trial database by pharmacists, research

For peer review only - http://bmjopen.bmj.com/site/about/guidelines.xhtml

nurses or staff at the trial co-ordinating centre. The data management and statistical support (including responsibility of data and final dataset) for the study is provided by the UKCRC registered Clinical Trial Unit (CTU) the Centre for Healthcare Randomised Trials (CHaRT) at the University of Aberdeen while the trial management is provided by another UKCRC registered CTU, the Edinburgh Clinical Trials Unit (ECTU) at Edinburgh University.

Ethics and dissemination

The Bridge-It trial involves procedures and medications which are well established in current NHS clinical practice and use. Adverse events may occur during or after the use of EC or POP and are well documented in the POP patient information leaflet. Serious adverse events will be recorded at the four month follow up interview and reported to the study sponsor. The study will be conducted in accordance with the principles of Good Clinical Practice (GCP).

A favorable ethical opinion has been obtained from the South East Scotland REC in June 2017. Approvals have been obtained from NHS Research Scotland (NRS) and Health Research Authority (HRA) England prior to commencement of the study. Annual progress reports and a final report at the conclusion of the trial will be submitted to REC within the timelines defined in the regulations. Protocol modifications are communicated by the ECTU to study sites via email and electronic newsletters.

The Bridge-It study website will include trial materials, trial progress, and summaries of key findings. In addition, public engagement and dissemination will also be undertaken via our Patient Public Involvement group.

The results of the study will be published in the academic journals and all participants will be offered a lay summary of the main findings of the study. The findings will also be presented at national and international conferences and disseminated via social media.

Trial Status

As at 7th Feb 2019 the study had recruited 503 participants across 29 sites. Recruitment to the first period completed on 13th Jan 2019, with 391 participants recruited at 29 sites. Recruitment is scheduled to be completed by Jun 2019, with analyses of the 4-month primary outcome expected to be available by Oct 2019.

DISCUSSION

Unintended pregnancy is widely perceived as a major public health problem. The proposed intervention in the Bridge-It study provides both temporary contraception (the POP) and facilitates access to effective contraception at a local SRH clinic. The cluster design was felt necessary for logistical reasons and confirmed in the qualitative work of our pilot study [17, 23] that an individually randomised trial would simply not recruit, as it was not feasible for pharmacists within a busy pharmacy to take additional time to randomise each individual. The crossover nature of the cluster design was chosen for efficiency, and by having a different set of women recruited at a pharmacy in the two different periods we avoided contamination by the participant. The purpose of the washout out period (was to minimise intervention effect carrying over from one period to another, as part of any contamination effect mediated by the pharmacist. With the cluster crossover design,

For peer review only - http://bmjopen.bmj.com/site/about/guidelines.xhtml

each cluster will act as its own control and fewer pharmacies are required than with a parallel cluster design.
If our proposed intervention works, then this could prevent more unintended pregnancies for more women. If the intervention is cost effective then it could have cost savings for the NHS.

REFERENCES.

1. Sedgh G, Singh S, Shah IH et al. Induced abortion: incidence and trends worldwide from 1995 to 2008. *Lancet*. 2012Feb 18;379(9816):625-32.

2.Department of Health. Abortion Statistics, England and Wales: 2017. https://www.gov.uk/government/publications/report-on-abortion-statistics-in-england-and-wales-for-2014 (access date 11 Jan 2019)

3. Information Services Division. Termination of Pregnancy Statistics 2017 http://www.isdscotland.org/Health-Topics/Sexual-Health/Abortions/ (access date 11 Jan 2019)

4. Wellings K, Jones KG, Mercer CH et al. The prevalence of unplanned pregnancy and associated factors in Britain: findings from the third National Survey of Sexual Attitudes and Lifestyles (Natsal-3). *Lancet* 2013;382:1807-16

5. Thomas C, Cameron ST. Can we reduce costs and prevent more unintended pregnancies? A cost of illness study illustrating the cost-saving potential of a more effective method of EHC. *BMJ open* 2013;3:e003815.doi:10.1136

6. SmithBattle L, Leonard V. Inequities compounded: explaining variations in the transition to adulthood for teen mothers' offspring. *J Fam Nurs*. 2012;18(3):409-31.

7. Cameron ST, Li H R**,** Gemzell –Danielsson K. Current controversies with Emergency Contraception: a review. *BJOG* 2017(13):1948-1956.

8. Marston C, Meltzer H, Majeed A. Impact on contraceptive practice of making emergency hormonal contraception available over the counter in Great Britain: repeated cross sectional surveys.*BMJ*. 2005Jul 30;331(7511):271.

9. Polis CB, Grimes DA, Schaffer K, et al . Advance provision of emergency contraception for pregnancy prevention. Cochrane Database of Systematic Reviews 2007,Issue2.Art. No: CD005497. DOI:0.1002/14651858.CD005497.pub2.

10. Faculty of Sexual and Reproductive Healthcare, UK. Emergency contraception. 2017. www.fsrh.org (access date 11 January 2019)

11. Cheng L, Che Y, Gülmezoglu AM. Interventions for emergency contraception. *Cochrane Database of Systematic Reviews* 2012;Issue 8. Art. No.: CD001324. DOI: 10.1002/14651858.CD001324.pub4.
12. Trussell J. Contraceptive efficacy. Global Library of Womens

For peer review only - http://bmjopen.bmj.com/site/about/guidelines.xhtml

Medicinehttp://www.glowm.com/section_view/heading/Contraceptive%20Efficacy/item/374 (access date 29 Jan 2019)

13 .Glasier A, Manners RJ, Loudon JC, et al. Community pharmacists providing emergency contraception give little advice about future contraceptive use: a mystery shopper study. *Contraception* 2010;82: 538–42.

14. The Faculty of Sexual and Reproductive Healthcare (FSRH). UK medical Eligibility criteria for Contraceptive Use. http://www.fsrh.org/standards-and-guidance/uk-medical-eligibility-criteria-for-contraceptive-use/ (access date 11 Jan 2019)

15. Brache V, Cochon L, Duijkers IJ, et al. A prospective, randomized, pharmacodynamic study of quick-starting a desogestrel progestin-only pill following ulipristal acetate for emergency contraception. *Hum Reprod* 2015;30(12):2785-2793

16. Cameron ST, Berger C, Michie L,et al. The effects on ovarian activity of 'quickstarting' a combined oral contraceptive pill after ulipristal acetate : prospective, randomised, double-blind parallel-arm, placebo-controlled study. *Hum Reprod* 2015;30(7):1566-1572.

17. Michie L, Cameron ST, Glasier A et al .Pharmacy based interventions for initiating effective contraception following the use of emergency contraception: a pilot study. *Contraception* 2014 ;90, 447-53

18. Arnup SJ, McKenzie JE, Hemming K, et al. Understanding the cluster crossover design: a graphical illustration of the components of variation and a sample size tutorial. *Trials.* 2017;18; 381.

19. Brueton VC, Tierney J, Stenning S, et al. Strategies to improve retention in randomised trials. Cochrane Database Syst Rev. 2013 Dec 3; 12:MR000032. doi: 10.1002/14651858.MR000032.pub2. 21

20. Cameron ST ,Glasier A. Identifying women in need of further discussion about the decision to have an abortion and eventual outcome. *Contraception*. 2013;88(1): 128-32

21. Turner RM, Omar RZ, Thompson SG. Modelling multivariate outcomes in hierarchical data, with application to cluster randomised trials. *Biom J*. 2006 Jun;48(3):333-45.

22. Ritchie J, Spencer L. Qualitative research practice: a guide for social science students and researchers. Sage: London, 2003.

23. Michie L, Cameron ST, Glasier A et al. Providing bridging contraception after emergency contraception from the pharmacy: a qualitative study. Public Health Public Health 2016; 135: 97-103.

For peer review only - http://bmjopen.bmj.com/site/about/guidelines.xhtml

Authors' contributions: STC, AG, JN, LMc D, AR, PB, JS, JT developed the original protocol. SC, CB, MF, RG, AMcD, BG, AJ, AM, SP,DS,NS, KC contributed to later stages of study design. STC wrote the draft of the protocol with significant input from all authors at all stages. All authors contributed, read and approved the final manuscript.

Author affiliations:

1. Chalmers Centre, NHS Lothian, Edinburgh EH3 9ES and University of Edinburgh
2. King's College Hospital NHS Foundation Trust, London, SE5 9RS
3. MRC/CSO Social & Public Health Sciences Unit, University of Glasgow, Glasgow G2 3AX
4. Edinburgh Clinical Trial Unit, Usher Institute, University of Edinburgh, Edinburgh EH16 4UX
5. King's Cross Hospital, NHS Tayside, Dundee DD3 8EA
6. University College London, EGA Institute for Women's Health, London WC1E 6AU
7. †Deceased December 2018. Princeton University USA.
8. Health Service Research Unit, University of Aberdeen, Aberdeen, AB25 2ZD
9. Boots UK, North Hub Office, Falkirk FK1 1ES
10. University College London, Institute for Global Health, London WC1E 6JB
11. King's College London, SE5 9RJ

Acknowledgements: Thank you to the members of the Patient and Public Involvement Group at the SRH service in Edinburgh who contributed to the design of this study. Thanks to the women who have participated in this study and to the community pharmacists, research staff and health care professionals at the local SRH services who have assisted with the implementation of this study. Thanks to Laura Flett for trial management support.

The Bridge-It Study Steering Committee provides oversight for this trial on behalf of the sponsor (University of Edinburgh and NHS Lothian) and the funder. The members are: Professor Peter Brocklehurst (Chair), Birmingham Clinical Trials Unit; Dr Lucy Michie, Sandyford Glasgow; Professor Kaye Wellings, London School of Hygiene and Tropical Medicine; Joanna Loudon, PPI member, Edinburgh; Kirsten Stuart PPI member Edinburgh; Emily Whittaker PPI member Edinburgh.

The Data Monitoring Committee is an independent multidisciplinary group consisting of clinicians and statisticians. The members are: Professor Claire Anderson (Chair), University of Nottingham; Professor Elizabeth Allen, London School of Hygiene and Tropical Medicine; Professor Caroline Moreau, Johns Hopkins Bloomberg School of Public Health, USA. A copy of the DMC charter is held in Edinburgh Clinical Trials Unit.

For peer review only - http://bmjopen.bmj.com/site/about/guidelines.xhtml

The study has co-sponsorship between The University Court of the University of Edinburgh and Lothian Health Board. The sponsors representative is accord@nhslothian.scot.nhs.uk

The paper presents independent research funded by the National Institute for Health Research (NIHR). The views expressed are those of the authors and not necessarily those of the NHS, the NIHR or the Department of Health.

Funding statement: The Bridge-It study is funded by the National Institute for Health Research's Health Technology Assessment Programme. HTA Project:15/113/01

Competing interests statement.

AG is a member of HRA Pharma scientific advisory board.

PB is a Clinical Director of the not-for profit community interest company SH:24 that provides online sexual health services in partnership with the NHS.

AR Research grants, educational grants and consultancy with Gilead
Research grants from Roche and BMS
Educational grants from Abbvie

JN is Deputy Chair of the NIHR/HTA General Board Committee. NIHR/HTA funded this research.

For peer review only

Table 1. Inclusion and exclusion criteria

**Inclusion Criteria**<br>• Intake of EC (levonorgestrel)<br>• Capacity to give informed consent to participate in the trial which includes adherence to trial requirements<br>• Age 16 years or over<br>• Willing to give contact details and be contacted at four months by phone or text or e-mail or post<br>• Willing to give identifying data sufficient to allow data linkage with NHS registries
**Exclusion Criteria**<br>• Contraindications to the POP<br>• On medication that interacts adversely with POP<br>• Already using a hormonal method of contraception<br>• Require interpreting services<br>• If pharmacist has concerns about non-consensual sex

For peer review only

Table 2. Study outcomes

		Data Source
Main outcome	Use of effective contraceptive method (hormonal or intrauterine) in intervention vs control at 4 months	Self reported (telephone or self completed survey) at 4 months
Secondary Outcomes	Numbers undergoing an abortion within 12 months intervention vs control	National abortion registries
	Economic evaluation	Incremental cost effectiveness ratio for pregnancies prevented

For peer review only

For peer review only - http://bmjopen.bmj.com/site/about/guidelines.xhtml

Table 3: Process evaluation methods and data

**Theory**
Theory of change model
**Study Team**
Pharmacy recruitment forms (study team members involved in recruitment will routinely record decision making contributing to pharmacy selection, including: number of contacts made; responses from potential pharmacists; rationales for inclusion/exclusion; and reasons for refusal)
**Pharmacists**
Participant observation of training & review of training and intervention materials<br><br>Recruitment monitoring forms (n=100% of pharmacists) & protocol adherence checklists (n=100% of pharmacists)<br><br>Follow-up semi-structured telephone interviews with pharmacists (n=27; one with each pharmacy involved).
**SRH Providers**
Semi-structured telephone interviews with SRH providers (n=12; with Service Manager, mix staff at 2x services in London; 1x service in Edinburgh; 1x service in Dundee).
**Participants**
Telephone questionnaire administered by Research Nurse at 4 months post-intervention (n=100% participants)<br><br>Semi-structured telephone interviews at 4 months post-intervention (n=60; 22 in London, 30 in Edinburgh and 8 in Dundee)
**Context**
Audit of local contraceptive services within 10 miles of study sites in London, Edinburgh and Dundee<br><br>Monitoring of contemporaneous events, such as relevant high coverage media stories using Google Alerts

For peer review only - http://bmjopen.bmj.com/site/about/guidelines.xhtml

Figure Legend

Figure 1. Bridge-it flowchart

POP= progestogen only pill
IDI= in depth interviews
SRH= sexual and reproductive health

For peer review only

For peer review only - http://bmjopen.bmj.com/site/about/guidelines.xhtml

Figure 1: Bridge-It study flowchart

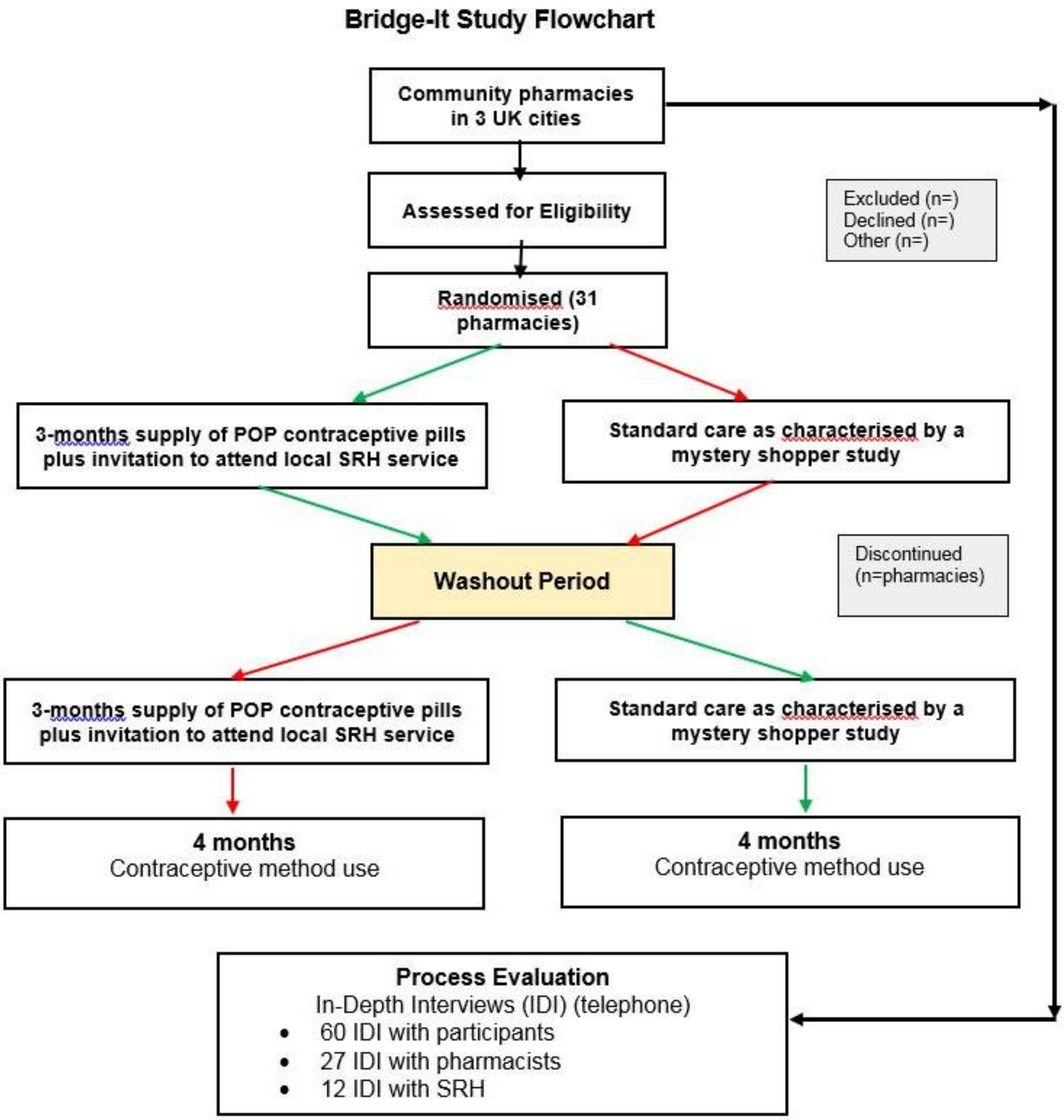

STANDARD PROTOCOL ITEMS: RECOMMENDATIONS FOR INTERVENTIONAL TRIALS

SPIRIT 2013 Checklist: Recommended items to address in a clinical trial protocol and related documents*

Section/item	Item No	Description: Bridge it study	Addressed on page number
**Administrative information**			
Title	1	Descriptive title identifying the study design, population, interventions, and, if applicable, trial acronym	__________1____
Trial registration	2a	Trial identifier and registry name. If not yet registered, name of intended registry	___________1___
	2b	All items from the World Health Organization Trial Registration Data Set	____________N/A_
Protocol version	3	Date and version identifier	_____1_________
Funding	4	Sources and types of financial, material, and other support	_______________1
Roles and responsibilities	5a	Names, affiliations, and roles of protocol contributors	________1,13_______
	5b	Name and contact information for the trial sponsor	_______13_______
	5c	Role of study sponsor and funders, if any, in study design; collection, management, analysis, and interpretation of data; writing of the report; and the decision to submit the report for publication, including whether they will have ultimate authority over any of these activities	___________13___
	5d	Composition, roles, and responsibilities of the coordinating centre, steering committee, endpoint adjudication committee, data management team, and other individuals or groups overseeing the trial, if applicable (see Item 21a for data monitoring committee)	_____________13_

For peer review only

Introduction

Background and rationale	6a	Description of research question and justification for undertaking the trial, including summary of relevant studies (published and unpublished) examining benefits and harms for each intervention	______________3, 4
	6b	Explanation for choice of comparators	_3_____________
Objectives	7	Specific objectives or hypotheses	__4____________
Trial design	8	Description of trial design including type of trial (eg, parallel group, crossover, factorial, single group), allocation ratio, and framework (eg, superiority, equivalence, noninferiority, exploratory)	2,4 ______________

Methods: Participants, interventions, and outcomes

Study setting	9	Description of study settings (eg, community clinic, academic hospital) and list of countries where data will be collected. Reference to where list of study sites can be obtained	_4_____________
Eligibility criteria	10	Inclusion and exclusion criteria for participants. If applicable, eligibility criteria for study centres and individuals who will perform the interventions (eg, surgeons, psychotherapists)	Table1__________ __
Interventions	11a	Interventions for each group with sufficient detail to allow replication, including how and when they will be administered	___5___________
	11b	Criteria for discontinuing or modifying allocated interventions for a given trial participant (eg, drug dose change in response to harms, participant request, or improving/worsening disease)	__N/A___________ _
	11c	Strategies to improve adherence to intervention protocols, and any procedures for monitoring adherence (eg, drug tablet return, laboratory tests)	_________6_____
	11d	Relevant concomitant care and interventions that are permitted or prohibited during the trial	__________N/A___ _
Outcomes	12	Primary, secondary, and other outcomes, including the specific measurement variable (eg, systolic blood pressure), analysis metric (eg, change from baseline, final value, time to event), method of aggregation (eg, median, proportion), and time point for each outcome. Explanation of the clinical relevance of chosen efficacy and harm outcomes is strongly recommended	___6___________

For peer review only - http://bmjopen.bmj.com/site/about/guidelines.xhtml

Participant timeline	13	Time schedule of enrolment, interventions (including any run-ins and washouts), assessments, and visits for participants. A schematic diagram is highly recommended (see Figure)	_____Fig1________
Sample size	14	Estimated number of participants needed to achieve study objectives and how it was determined, including clinical and statistical assumptions supporting any sample size calculations	_________8____
Recruitment	15	Strategies for achieving adequate participant enrolment to reach target sample size	_________6____

Methods: Assignment of interventions (for controlled trials)

Allocation:

Sequence generation	16a	Method of generating the allocation sequence (eg, computer-generated random numbers), and list of any factors for stratification. To reduce predictability of a random sequence, details of any planned restriction (eg, blocking) should be provided in a separate document that is unavailable to those who enrol participants or assign interventions	____6_________
Allocation concealment mechanism	16b	Mechanism of implementing the allocation sequence (eg, central telephone; sequentially numbered, opaque, sealed envelopes), describing any steps to conceal the sequence until interventions are assigned	_________6____
Implementation	16c	Who will generate the allocation sequence, who will enrol participants, and who will assign participants to interventions	____6 _N/A as cluster randomised________
Blinding (masking)	17a	Who will be blinded after assignment to interventions (eg, trial participants, care providers, outcome assessors, data analysts), and how	__N/A___________
	17b	If blinded, circumstances under which unblinding is permissible, and procedure for revealing a participant's allocated intervention during the trial	_____N/A________

Methods: Data collection, management, and analysis

Data collection methods	18a	Plans for assessment and collection of outcome, baseline, and other trial data, including any related processes to promote data quality (eg, duplicate measurements, training of assessors) and a description of study instruments (eg, questionnaires, laboratory tests) along with their reliability and validity, if known. Reference to where data collection forms can be found, if not in the protocol	_________7,9_____
	18b	Plans to promote participant retention and complete follow-up, including list of any outcome data to be collected for participants who discontinue or deviate from intervention protocols	___________6__
Data management	19	Plans for data entry, coding, security, and storage, including any related processes to promote data quality (eg, double data entry; range checks for data values). Reference to where details of data management procedures can be found, if not in the protocol	____________9_
Statistical methods	20a	Statistical methods for analysing primary and secondary outcomes. Reference to where other details of the statistical analysis plan can be found, if not in the protocol	__________8,9
	20b	Methods for any additional analyses (eg, subgroup and adjusted analyses)	_8,9____________
	20c	Definition of analysis population relating to protocol non-adherence (eg, as randomised analysis), and any statistical methods to handle missing data (eg, multiple imputation)	_8____________

Methods: Monitoring

Data monitoring	21a	Composition of data monitoring committee (DMC); summary of its role and reporting structure; statement of whether it is independent from the sponsor and competing interests; and reference to where further details about its charter can be found, if not in the protocol. Alternatively, an explanation of why a DMC is not needed	_13____________
	21b	Description of any interim analyses and stopping guidelines, including who will have access to these interim results and make the final decision to terminate the trial	____N/A__________
Harms	22	Plans for collecting, assessing, reporting, and managing solicited and spontaneously reported adverse events and other unintended effects of trial interventions or trial conduct	_________10____
Auditing	23	Frequency and procedures for auditing trial conduct, if any, and whether the process will be independent from investigators and the sponsor	__N/A____________

Ethics and dissemination

Research ethics approval	24	Plans for seeking research ethics committee/institutional review board (REC/IRB) approval	________2______
Protocol amendments	25	Plans for communicating important protocol modifications (eg, changes to eligibility criteria, outcomes, analyses) to relevant parties (eg, investigators, REC/IRBs, trial participants, trial registries, journals, regulators)	_________10_____
Consent or assent	26a	Who will obtain informed consent or assent from potential trial participants or authorised surrogates, and how (see Item 32)	__________6____
	26b	Additional consent provisions for collection and use of participant data and biological specimens in ancillary studies, if applicable	___N/A__________
Confidentiality	27	How personal information about potential and enrolled participants will be collected, shared, and maintained in order to protect confidentiality before, during, and after the trial	_____This is in ethical application-on bridge it website _____
Declaration of interests	28	Financial and other competing interests for principal investigators for the overall trial and each study site	14 Also BMJ open IJMEC forms________
Access to data	29	Statement of who will have access to the final trial dataset, and disclosure of contractual agreements that limit such access for investigators	__page 9
Ancillary and post-trial care	30	Provisions, if any, for ancillary and post-trial care, and for compensation to those who suffer harm from trial participation	__ PIS, and consent__uploaded as supplementary__________
Dissemination policy	31a	Plans for investigators and sponsor to communicate trial results to participants, healthcare professionals, the public, and other relevant groups (eg, via publication, reporting in results databases, or other data sharing arrangements), including any publication restrictions	__10___________

For peer review only - http://bmjopen.bmj.com/site/about/guidelines.xhtml

	31b	Authorship eligibility guidelines and any intended use of professional writers	____12___________
	31c	Plans, if any, for granting public access to the full protocol, participant-level dataset, and statistical code	_________N/A_____ _
**Appendices**			
Informed consent materials	32	Model consent form and other related documentation given to participants and authorised surrogates	__uploaded as supplementary files<br>Participant consent form<br>Participant information sheet<br>Pharmacist/SRH provider information sheet<br>Pharmacist/SRH provided consent form<br>GDPR participant Information sheet
Biological specimens	33	Plans for collection, laboratory evaluation, and storage of biological specimens for genetic or molecular analysis in the current trial and for future use in ancillary studies, if applicable	N/a

*It is strongly recommended that this checklist be read in conjunction with the SPIRIT 2013 Explanation & Elaboration for important clarification on the items. Amendments to the protocol should be tracked and dated. The SPIRIT checklist is copyrighted by the SPIRIT Group under the Creative Commons "Attribution-NonCommercial-NoDerivs 3.0 Unported" license.

For peer review only
